# BUB1 an Overexpressed Kinase in Sarcoma: Finding New Target Therapy for Osteosarcoma, Liposarcoma, Synovial Sarcoma, and Leiomyosarcoma

**DOI:** 10.3390/biom15071046

**Published:** 2025-07-18

**Authors:** Mercedes Olvera-Valencia, Fernando Luna-Maldonado, Joselyn Juarez-Reyes, Alejandro Lopez-Saavedra, Jossimar Coronel-Hernandez, Oliver Millan-Catalan, Daniel Guzman-Gomez, Frida Rodríguez-Izquierdo, Luis A. Herrera, David Francisco Cantú-De León, Carlos Perez-Plasencia, Eloy-Andres Pérez-Yepez

**Affiliations:** 1 Programa Institucional de Biomedicina Molecular, Escuela Nacional de Medicina y Homeopatía del IPN, Guillermo Massieu Helguera #239 Fracc. La escalera, Ticoman 07320, Ciudad de Mexico, Mexico; mercedesvalol@gmail.com; 2 Laboratorio de Genómica, Instituto Nacional de Cancerología, San Fernando 22. Col. Sección XVI, Tlalpan 14080, Ciudad de Mexico, Mexico; zs20007612@estudiantes.uv.mx (J.J.-R.); jossithunders@gmail.com (J.C.-H.); oliver.millan.sg@gmail.com (O.M.-C.); fridaizquierdo19@gmail.com (F.R.-I.); 3 Unidad de Investigación Biomédica en Cáncer, Insituto de Investigaciones Biomédicas-Universidad Nacional Autónoma de México, Instituto Nacional de Cancerología, Mexico City 14080, Mexico; ferlmgp@gmail.com (F.L.-M.); metil@hotmail.com (L.A.H.); 4 LADISER de Inmunología y Biología Molecular, Facultad de Ciencias Químicas, Universidad Veracruzana (UV), Prolongación de Oriente 6 #1009, Colonia Rafael Alvarado, Orizaba 94340, Veracruz, Mexico; daguzman@uv.mx; 5 Advanced Microscopy Applications Unit (ADMIRA)-Instituto Nacional de Cancerología, San Fernando 22. Col. Sección XVI, Tlalpan 14080, Ciudad de Mexico, Mexico; alexlosaa@tec.mx; 6 Tecnológico de Monterrey, Escuela de Medicina y Ciencias de la Salud, Ciudad de México 14380, Mexico; 7 Experimental Biology Program, DCBS, Universidad Autónoma Metropolitana-Iztapalapa, Ciudad de Mexico 09340, Mexico; 8 Clinical Research, Instituto Nacional de Cancerologia, San Fernando 22. Col. Sección XVI, Tlalpan, Ciudad de Mexico 14080, Mexico; dfcantu@gmail.com; 9 Laboratorio de Genómica Funcional, Unidad de Biomedicina, Facultad de Estudios Superiores Iztacala, UNAM, Tlalnepantla Estado de México 54090, Mexico

**Keywords:** sarcoma, kinases, bone sarcoma, therapeutic target, soft tissue sarcomas

## Abstract

Sarcomas are heterogeneous mesenchymal tumors, and their pharmacological treatment remains challenging due to the high toxicity and poor efficacy of current therapies. This study aimed to identify common overexpressed kinases in the four most frequent sarcoma subtypes to establish novel therapeutic targets. A bioinformatics approach using patient-derived gene expression data sets identified overexpressed kinases shared across these sarcoma types. Later, *BUB1* was determined as the kinase consistently overexpressed across the osteosarcoma, liposarcoma, leiomyosarcoma, and synovial sarcoma. Moreover, the role of this kinase was further validated through molecular and functional assays, including pharmacological inhibition in cell lines derived from the four sarcoma subtypes. BUB1 inhibition reduced the phosphorylation of AKT and H2A proteins, precluded cell proliferation, and inhibited colony formation in sarcoma cells. Finally, overall survival analysis highlighted a strong correlation between high *BUB1* expression and poorer survival rates in sarcoma patients. Altogether, these findings underscore the potential of BUB1 as a therapeutic target and prognostic marker in sarcomas. Targeted inhibition of BUB1 may provide a novel strategy to reduce tumor growth and improve outcomes for patients with bone and soft tissue sarcomas.

## 1. Introduction

Sarcomas is a heterogeneous group of neoplasms derived from mesenchymal cells originating from the mesodermal germ layer [1]. There are approximately 50 different histological varieties of sarcomas; for study purposes, they are defined into two large groups: soft tissue sarcoma (STS) and bone tissue sarcoma (BS), which represent 80% and 20% of tumors, respectively [2]. The most frequent STS subtypes are liposarcoma, leiomyosarcoma, dedifferentiated pleomorphic, and synovial sarcoma [3]. While the most common BS are osteosarcomas (OS), chondrogenic, fibro genic, fibro histiocytic, and Ewing [4]. Sarcoma affects a vulnerable population, including children, adolescents, and economically active young adults [5,6]. The therapeutic objectives for sarcoma are to reduce recurrence and minimize morbidity and mortality. In patients with high-grade tumors, multimodal therapy together with surgery and radiotherapy has been shown to minimize the incidence of recurrent disease and is associated with an increase in overall survival of up to 10% [7]. For the past 40 years, cytotoxic agents led by anthracyclines, alkylating agents, and taxanes have remained as standard treatment for sarcomas. In metastatic disease, doxorubicin and ifosfamide are first-line, cytotoxic, antineoplastic drugs with a response rate of around 20–30% [8]. Gemcitabine alone or in combination with docetaxel is the standard second-line treatment. Eribulin has recently been approved for patients with liposarcomas, and trabectedin is approved for patients with leiomyosarcoma and liposarcomas [9]. In the case of BS, methotrexate, doxorubicin, and cisplatin (MAP) are the standard treatment in North America and Europe, with response rates in 70% of treated patients [10]. It has been known that a problem in the treatment of sarcomas is the resistance to conventional chemotherapeutic agents [11]. For example, it is estimated that about 30–40% of patients with osteosarcoma who receive systemic treatment develop resistance [12]. It is known that a wide number of cellular mechanisms lead to cancer chemo-resistance such as the expression of ABC transporters, alterations in the apoptosis mechanism, overexpression, and hyperactivation of kinases and phosphatases [11], among others.

Protein phosphorylation is driven by kinases, and the dysregulation of this process plays a fundamental role in cell proliferation, migration, and differentiation during cancer progression [13]. Recently, several clinical trials using Tyrosine Kinase Inhibitors (TKIs) or antibodies to block the kinase activity of receptors have been carried out for sarcomas [14]. The inhibitors administered in combination or concomitance with systemic drugs, such as doxorubicin, showed the best results compared to standalone treatments. Also, the kinase inhibition downstream of receptors such as mTORC has shown promissory results as antitumor agents in these tumors [15]. However, few TKIs, such as imatinib, pazopanib, or olaratumab, are approved by the FDA for clinical use in sarcoma patients [14]. This could be due to the RTK’s activity depending on the sarcoma subtype [16]. For these reasons, the analysis of the axis phosphorylation/dephosphorylation by establishing enzymes that drive it in sarcomas may be key to improving new therapeutic schemes.

The serine/threonine kinase BUB1 is essential for the mitotic spindle checkpoint and chromosome alignment during mitosis [17]. This protein, together with Mad1–3p, Bub1p, Bub3p, and TKK, regulates chromosome segregation [18]. BUB1 activity has been associated with cancer progression through cell proliferation, migration, and epithelial–mesenchymal transition (EMT) activation. Moreover, its high-expression levels are related to worse overall survival (OS) of patients with several types of cancer as colon, lung, breast, melanoma, and osteosarcoma [18,19,20].

In this work, we used gene expression databases and bioinformatic tools to identify the overexpressed kinases in the four most frequent types of sarcomas: osteosarcoma, synovial sarcoma, liposarcoma, and leiomyosarcoma. Then, overexpressed (OE) kinases shared in all analyzed sarcomas were determined. BUB1 kinase was identified as a key enzyme across all four types of sarcomas analyzed. The interaction network for BUB1 expression regulation was predicted and a gene set enrichment analysis was performed to determine biological implications. BUB1 interactions suggest a potential role in promoting cell proliferation and invasion. Signaling pathways that regulate malignancy and progression of STS and BS were the most represented. Using sarcoma cell lines and non-tumoral mesenchymal cells, the gene expression and protein levels of BUB1 were measured. Pharmacological BUB1 inhibition assays showed an implication in cell proliferation of this kinase. Finally, a correlation between high levels of BUB1 and poor overall survival in patients of all analyzed sarcomas was determined. All this evidence allows us to propose BUB1 as a key kinase in the progression of OS, liposarcoma, leiomyosarcoma, and synovial sarcoma. The inhibition of BUB1 through targeted therapy not only represents a novel pharmacologic strategy for sarcoma treatment but may also contribute to improving patient outcomes by limiting tumor proliferation and invasion. Future research should focus on validating these findings in clinical settings and exploring combination therapies to enhance the efficacy of BUB1 inhibition.

## 2. Materials and Methods

### 2.1. Patient Data

To identify overexpressed kinases in the four types of sarcomas analyzed in this work, we developed a bioinformatics workflow utilizing several data mining methods implemented through the R programming language (4.1.1), the Bioconductor environment (3.13), and gene expression data sets [21]. We obtained gene expression data from tumor tissue of osteosarcoma, liposarcoma, leiomyosarcoma, and synovial sarcoma in the Gene Expression OSmnibus repository. Each data set contains the global expression of both tumor and adjacent normal tissue. Table 1 enlists the GEO accession number, sequencing technology, access link, and information about the project for each type of sarcoma used.

### 2.2. Kinase Expression Determination

To determine which of the transcripts of the database correspond to kinases, we used the KinHub tool [22] to obtain a list of genes that codify the human kinome (536 kinases) to perform the intensity expression in each sarcoma type.

### 2.3. Differential Expression Analysis

To establish the differentially expressed (DE) kinase genes in each type of sarcoma, we employed the DESeq2 package [23] and loaded the expression matrix and the sample metadata (i.e., whether samples were healthy or tumoral) into this package. Fold changes were calculated by comparing gene expression levels. Genes with a fold change threshold > 1.0 were considered differentially expressed based on the adjusted *p*-value (<0.05). DE genes were normalized using DESeq2. The R package (version number 2024.12.1) ggplot was used for volcano plot visualization. Finally, we developed a comparative analysis using the VennDiagram R package [24] to identify the overexpressed (OE) kinases shared across the different sarcoma types.

### 2.4. BUB1 mRNA Interaction Predictions

BUB1 transcriptional regulation was analyzed, first determining its interaction with gene expression regulatory molecules such as transcription factors, mRNA-interacting molecules, and non-coding RNAs (microRNAs and long non-coding RNAs). Then MiRTarBase [25], a database that compilated the interaction data of mRNA-miRNAs with experimental evidence, was tested. Finally, the BUB1 interactions with transcription factors, RNA-binding proteins, and long non-coding RNAs were tested using RNAinter [26]. The interactions with a confidence score higher than 0.4 were considered for further analysis. With all the output data, we perform an interactome by cystoscape software (Version 3.10.1). Finally, to analyze the effect of the interaction of mRNA interaction protein, miRNA-BUB1 mRNA, or LncRNA-BUB1 mRNA on the cancer phenotype, we performed an enriched signaling pathways and biological processes test. MiRNET and ShinyGO2.0 enrichment tools (version number 0.81) were used, respectively.

### 2.5. Sarcoma Cell Lines and Non-Tumoral Mesenchymal Cells Culture

The cell lines HTB-86 (U-2 OS), HTB-92 (SW-872), HTB-88 (SK-LMS-1), and HTB-93 (SW-982) derived from osteosarcoma, liposarcoma, leiomyosarcoma, and synovial sarcoma, respectively, were obtained from ATCC (Manasas, VA, USA). Cell cultures were handled following the provider’s specifications and the indicated cell culture media. Each cultured media was supplemented with 10% fetal bovine serum (ATCC-30-2021), 100 U/mL of penicillin, and 100 μg/mL of streptomycin (ATCC-30-2300). Cultures were incubated at 37 °C with 5% CO_2_ for HTB-86 and HTB-88, whereas HTB-92 and HBT-93 cell lines were incubated at 37 °C in 100% air atmosphere.

Non-tumoral mesenchymal cells correspond to Dental Pulp Stem Cells (DPSCs) obtained from the first molar of a routine exodontia in a 32-year-old, apparently healthy female patient with her informed consent. Isolation was performed as previously reported by several authors [27,28]. Briefly, the dental specimen was maintained in a physiological solution at 4 °C after extraction and before digestion to preserve the cellular content. The dental pulp was isolated and immersed in a digestion solution (3 mg/mL collagenase type I/dispase in DMEM-F12). After incubation for 45 min at 37 °C, with shaking every 10 min, the enzymes were inactivated with 10% SFB and resuspended in fresh media, the cell suspension was placed in 25 cm^2^ flask in culture medium (DMEM-F12 with 10% inactivated fetal bovine serum, 2 mM L-glutamine, 100 U/mL penicillin, and 100 mg/mL streptomycin), and the flask was placed in an incubator at 37 °C and 5% CO_2_. DPSCs were expanded until they reached 70% confluence. Cell passages were performed with 0.05% trypsin-EDTA. Cryopreservation was carried out in complete DMEM-F12 medium plus 10% DMSO at −70 °C.

### 2.6. RNA Purification and RT-qPCR

Total RNA was extracted using Trizol^®^ reagent (Invitrogen, Carlsbad, CA, USA). One microgram of total RNA was used for reverse transcriptase reactions. Gene expression was carried out using the SYBR Green Master kit (Thermo-Fisher, Waltham, MA, USA) for real-time PCR and BUB1-specific primers (Forward: 5′-AGCCCAGACAGTAACAGACTC-3′ Reverse: 5′-GTTGGCAACCTTATGTGTTTCAC-3′; Tm: 60 °C). Relative gene expression values were normalized to the constitutive expression of beta-actin (Forward: 5′-ATGACTTAGTTGCGTTACACCCT-3′ Reverse: 5′-TGCTCGCTCCAACCGACTG-3′; TA: 60 °C). Fold change was determined using the 2^−ΔΔCT^ method.

### 2.7. Total Protein Extraction and Western Blot Analysis

Protein extracts were obtained by cell lysis with Pierce^®^ RIPA buffer (Thermo Scientific, Waltham, MA, USA), complemented with Complete^®^ 1X, and quantified by micro-Bradford. Proteins were SDS-PAGE-separated in 10–15% gels and blotted onto PVDF membranes blocked with 5% non-fat milk/TBS-0.1% Tween 20. Membranes were exposed to mouse anti-human BUB1 (Cat. Sc-47743, Santa Cruz Biotechnology, Dallas, TX, USA), rabbit anti-phospho Ser473 AKT (Cat. 9271, Cell Signaling, Danvers, MA, USA), rabbit anti-total AKT (Cat. 9272S, Cell Signaling, Danvers, MA, USA), rabbit anti-phospho Thr120 Histone 2A (Cat. 39391, Active Motif, Carlsbad, CA, USA), rabbit anti-total Histone 2A (Cat. 700158, Thermo-Fisher, Waltham, MA, USA), or mouse anti-β-actin (Cat. sc-47778, Santa Cruz Biotechnology, Dallas, TX, USA) antibodies. Secondary antibodies used were HRP-labeled anti-rabbit (Cat. 7074S) or anti-mouse (Cat. 7076S) IgG (both from Cell Signaling, Danvers, MA, USA) and bands revealed by chemiluminescence. Densitometry analyses were performed with ImageJ software (Version 1.54i).

### 2.8. BUB1 Pharmacological Inhibition

To inhibit BUB1 kinase activity, the sarcoma cell lines were treated with the inhibitor 2OH-BNPP1 (Cat. HY-102081, MedChemExpress, Monmouth Junction, NJ, USA). For that, 15,000 cells/cm^2^ were seeded and, 24 h later, were treated with the inhibitor at 5 µM, 10 µM, and 20 μM. After 24 h of treatment, the cells were harvested to proceed with analysis. As controls, non-treated cells (NT) or cells treated with DMSO (vehicle control) were used.

### 2.9. Colony Formation Assay

After 24 h of treatment with 2OH-BNPP1 inhibitor, cells were cultured and seeded in 6-well plates (500 cells/well). After ten days of culture, cells were fixed with 6% glutaraldehyde for thirty minutes and stained with 1% crystal violet for ten minutes. The colonies were counted using the ImageJ software (version number 1.54g).

### 2.10. Proliferation Assay

Briefly, cells were seeded in a 96-well plate and treated with BUB1 inhibitor as described at Section 2.8 for 24 h. Cell viability and proliferation were tested 24 h later using the MTT assay. Cells were washed with PBS and exposed to MTT (Sigma-Aldrich, St. Louis, MO, USA) for 3 h at 37 °C. Then, they were washed and incubated with 100 μL DMSO for 15 min. The optical density (OD) was recorded at 540 nm in an Epoch microplate spectrophotometer (Biotek, Winooski, VT, USA).

### 2.11. Overall Survival Analysis

The correlation between the BUB1 expression and the clinical outcome of sarcoma patients was explored by survival analysis with the survival package in R by Kaplan–Meir analysis [29]. We assessed statistical significance using a log-rank and cumulative hazard test with Cox regression.

### 2.12. Statistical Analysis

Data are expressed as the mean ± the standard deviation of three independent experiments. *p*-values less than 0.05 were considered statistically significant and are represented as *p* < 0.05 *, *p* < 0.01 **, and *p* < 0.001 ***. All statistical analyses were performed in the GraphPad Prism 6 software.

## 3. Results

### 3.1. Kinase Expression Profile Establishment in Osteosarcoma, Liposarcoma, Synovial Sarcoma, and Leiomyosarcoma

**Figure 1:**
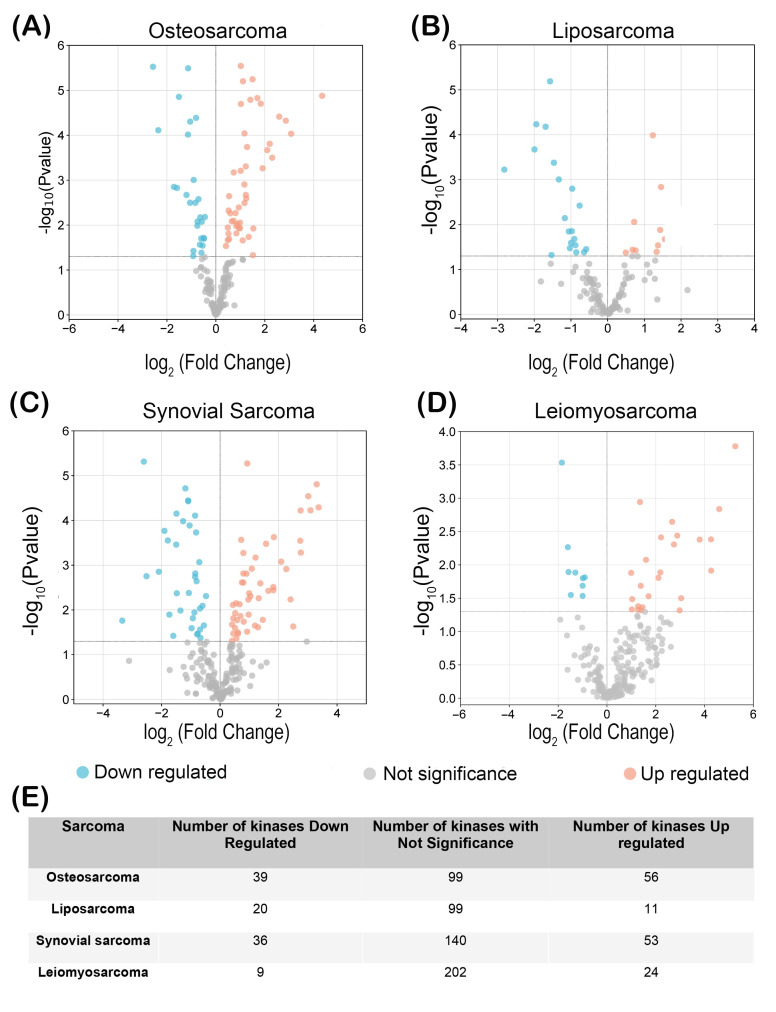
Differential expression of kinases in sarcoma tissues. Volcano plot graphs show the relative expression of kinases in analyzed sarcomas (**A**) osteosarcoma, (**B**) liposarcoma, (**C**) synovial sarcoma, and (**D**) leiomyosarcoma. (**E**) Numbers of downregulated, no significantly changed, and upregulated kinases in the sarcomas.

Global expression data from patient samples of osteosarcoma, liposarcoma, synovial sarcoma, and leiomyosarcoma available in the GEO (Gene Expression Ommnibus) repository were collected (Table 1). These data were filtered to determine the expression levels of 536 kinases. Using the DEseq2 statistical package (version number 1.42.1), kinase expression changes in tumor tissues were compared versus the expression of the same kinase in adjacent healthy or non-tumor tissues for each type of sarcoma analyzed. The kinases were grouped according to expression levels as low expression, not sig (no expression changes), and high expression. Volcano plots in Figure 1 show the fourth groups for osteosarcoma (A), liposarcoma (B), synovial sarcoma (C), and leiomyosarcoma (D). In Figure 1E, the number of kinases grouped for each type of sarcoma is shown. For this study, the overexpressed (OE) kinases were the group of interest. For osteosarcoma, 56 kinases were grouped as OE, whereas liposarcoma, synovial sarcoma, and leiomyosarcoma were grouped as 11, 53, and 24 kinases, respectively. On the other hand, the kinases with low expression were 39 for osteosarcoma, 20 for liposarcoma, 36 for synovial sarcoma, and only 9 for leiomyosarcoma.

### 3.2. BUB1 Kinase Overexpression Correlates in Osteosarcoma, Liposarcoma, Synovial Sarcoma, and Leiomyosarcoma

**Figure 2:**
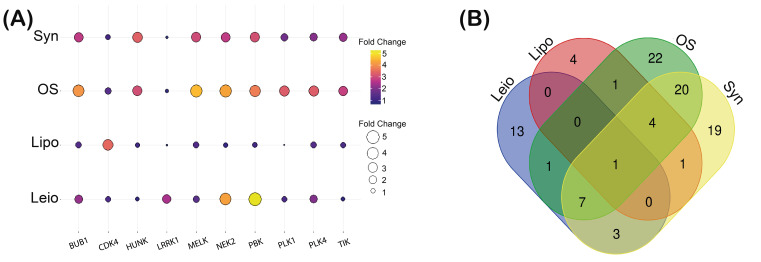
Overexpressed kinases that are shared by the 4 types of sarcomas studied. (**A**) Balloon plot that shows the top ten kinases with high expression levels of each sarcoma type; size and color represent the fold change in expression for each one. (**B**) Venn diagram comparing the overexpressed kinases in osteosarcoma (green), synovial sarcoma (yellow), liposarcoma (red), and leiomyosarcoma (purple). The kinases that match between the different types of sarcomas are counted within the intersections of each color. Syn (synovial sarcoma); Os (osteosarcoma); Lipo (liposarcoma); Leio (leiomyosarcoma).

Once we determined the profile of OE kinases in the sarcomas, we performed a clustering analysis to identify kinases that shared high expression levels in the four sarcomas studied. Figure 2A displays a ballon plot that enlists the top ten OE kinases for each type of sarcoma. The size and color of each balloon represent the fold change in the expression levels for each kinase (comparing sarcoma vs. non-tumoral tissues). BUB1, CDK4, HUNK, LRRK1, MELK, NEK2, PBK, PLK1, PLK4, and TTK were established as the most overexpressed across the four sarcomas. To determine overexpressed kinases that were shared between the sarcomas, a comparative analysis was performed. Figure 2B shows a Venn diagram that represents the overexpressed kinases (*p* < 0.05) shared between each sarcoma type. Interestingly, only one kinase is shared among the four subtypes analyzed. This kinase is called BUB1, which is a serine/threonine-type kinase that plays a crucial role during mitosis, and alterations in its expression levels have been associated with cancer progression [30]. This data suggests BUB1 as a nodal kinase for all the four sarcomas analyzed.

### 3.3. The Network of Regulation of BUB1 Is Associated with Cancer Progression Signaling Pathways

**Figure 3:**
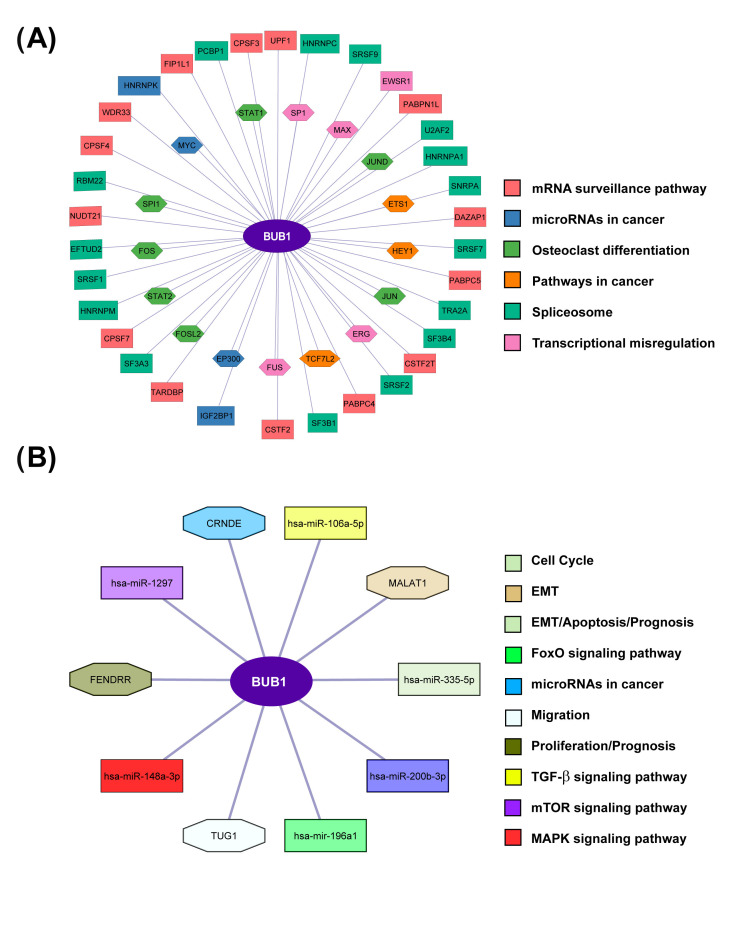
BUB1 mRNA interaction network and its relation to biological functions. (**A**) The regulation of BUB1 expression levels by transcription factors (hexagons) or RNA-binding proteins (rectangles) are schematized. (**B**) The interaction of BUB1 mRNA with non-coding RNAs (lncRNAs in octagons and miRNAs in rectangles) is shown. Each interaction component is colored according to the biological function or signaling pathway in which it participates.

To determine the biological implications of BUB1 expression levels in sarcomas, interaction analyses were developed. Its transcriptional regulation was established by determining BUB1 interactions with gene expression regulators such as transcription factors, RNA-binding proteins, and non-coding RNAs (long non-coding and microRNAs) (Figure 3). Transcription factors such as STAT1, STAT2, FOS, MYC, E2F4, EP300, and JUN could regulate the expression levels of BUB1. Additionally, the interaction of BUB1 mRNA with RNA-binding proteins that trigger the transcription activity as SRSF family proteins suggests an important role of the BUB1 expression in cancer progression. A gene set enrichment analysis using the proteins that interact with BUB1 showed that MRAN surveillance pathway, microRNAs in cancer, osteoclast differentiation, pathways in cancer, splicesome, and transcriptional misregulation in cancer could be the pathways involved in BUB1 overexpression. In addition, non-coding RNAs (ncRNAs) also interact with BUB1 mRNA. The long non-coding RNAs (LncRNAs) CRNDE, TUG1, MALAT1, and FENDRR, as well as the microRNAs (miRNAs) has-miR-106a-5p, hsa-mir-1297, has-miR-335-5p, hsa-miR-200b-3p, has-miR-196a1, and hsa-miR-148a-3p, are susceptible to regulation of the levels of BUB1. The enrichment analysis of the ncRNAs reveals cell cycle, epithelial–mesenchymal transition (EMT), FoxO signaling pathway, MAPK signaling pathway, migration, proliferation, TGF-Beta signaling pathway, and mTOR signaling pathway as the most enriched. All this evidence highlighted the participation of BUB1 expression in signaling pathways and cellular processes altered in cancer and positioned this kinase as a central molecule for malignancy in the sarcomas analyzed.

### 3.4. High Levels of BUB1 Kinase Are Shown in Several Types of Sarcomas, and Its Inhibition Precludes Cell Proliferation

**Figure 4:**
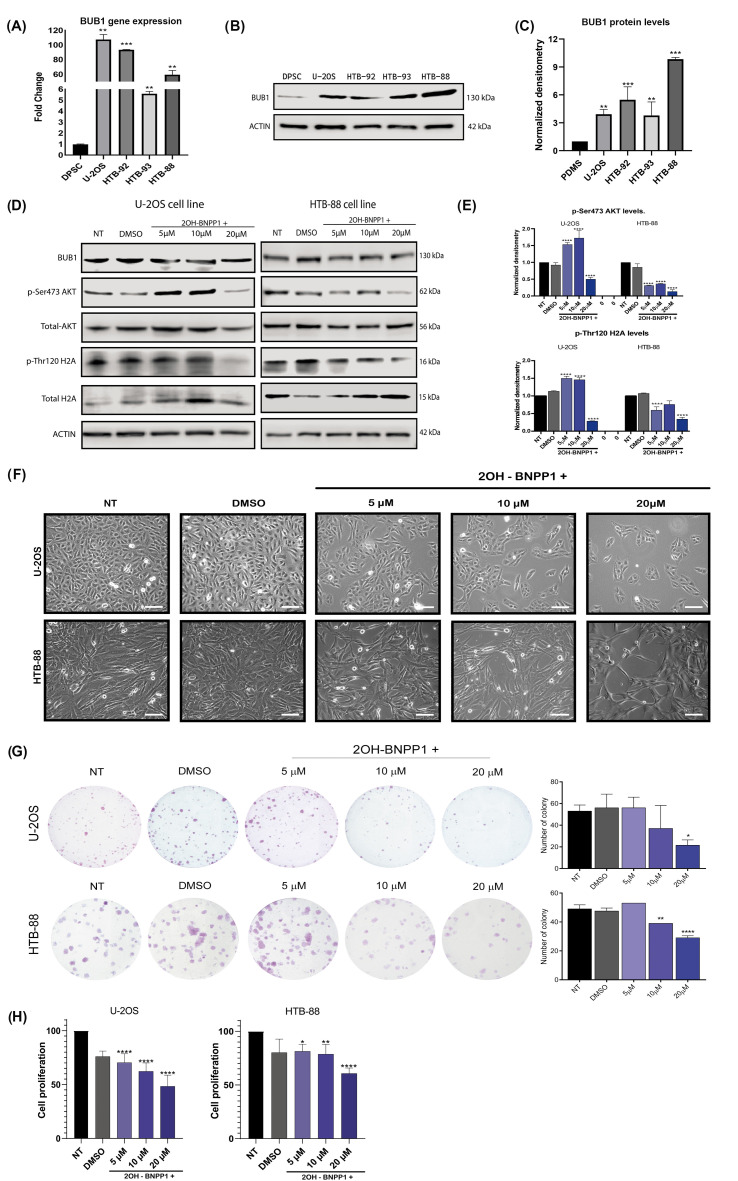
BUB1 levels in sarcoma cell lines and their effect on cell proliferation. (**A**) Gene expression levels and (**B**) protein levels of BUB1 in U-2OS (osteosarcoma), HTB-92 (liposarcoma), HTB-93 (synovial sarcoma), and HTB-88 (leiomyosarcoma) cell lines. Non-transformed mesenchymal cells (DPSC) were used as a control. (**C**) Densitometric analysis of three independent experiments of BUB1 protein levels in the different sarcoma cell lines. (**D**) Detection of Ser473-AKT and Thr120-Histone H2A phosphorylations in U-2OS and HTB-88 cells treated with 2-OH-BNPP1 at 5 mM, 10 mM, and 20 mM. (**E**) Densitometry of p-Ser473 AKT and p-Thr120 H2A protein levels in U-2OS and HTB-88 cells treated with 2-OH-BNPP1. (**F**) Widefield microscopy of osteosarcoma (U-2OS) and leiomyosarcoma (HTB-88) cells treated at different concentrations of the BUB1 kinase activity inhibitor 2-0H-BNPP1. Bar = 20 μm. (**G**) Colony formation assay comparing untreated and 2-OH-BNPP1-treated cells at different concentrations in U-2OS and HTB-88 cells. (**H**) Proliferation assay results of cells U-20S and HTB-88 treated with the BUB1 activity inhibitor. For (**G**) and (**H**), data are expressed as the mean ± the standard deviation of three independent experiments. * (*p* ≤ 0.05) ** (*p* ≤ 0.01) *** (*p* ≤ 0.001) **** (*p* ≤ 0.0001). NT (Not Treated).

To validate the high expression levels of BUB1, its gene expression and protein levels were evaluated using cell lines derived from osteosarcoma (U-2OS), liposarcoma (HTB-92), synovial sarcoma (HTB-93), and leiomyosarcoma (HTB-88). Dental pulp stem cell (DPSCs) were used as mesenchymal, non-transformed cells. BUB1 gene expression was significantly higher in U-2OS, HTB-92, HTB-93, and HTB-88 cells of 100-, 96-, 5-, and 60-fold, respectively, compared with gene expression levels in non-tumoral mesenchymal cells (Figure 4A). In addition, protein levels were also higher in sarcoma cells compared with the protein levels in PDSCs. Figure 4B shows a representative BUB1 Western blot assay. The densitometric analysis of three independent experiments revealed BUB1 levels up to 3, 5, and 9 times higher in sarcoma cell lines compared with the protein level in non-tumoral cells (Figure 4C). These findings are consistent with the patterns observed in tissue samples.

To determine the biological effect of BUB1 in sarcomas, we performed a pharmacologic inhibition of the activity of this kinase using the inhibitor 2-OH-BNPP1, which was previously described to preclude the kinase activity of BUB1 and block the EGFR and TGF-β signaling pathways [31,32]. Based on these reports, the cell lines U-2OS and HTB-88 (which showed the highest BUB1 protein levels) were treated at 5 μM, 10 μM, and 20 μM of the inhibitor. Later, phosphorylations of serine in position 473 of AKT (Ser473-AKT) and threonine 120 of histone 2A (Thr120-H2A), both previously reported targets of phosphorylation by BUB1 [33,34,35,36,19], were evaluated by Western blot. Treatment with 20 μM resulted in lower phosphorylation of both protein residues compared to untreated (NT) or vehicle-treated (DMSO) cells (Figure 4D,E), whereas the total protein levels for both targets or total-BUB1 did not change. It has been known that BUB1 regulates mitosis; for this reason, we explored the cellular growth of sarcoma cell lines in the presence of the kinase inhibitor. Figure 4F shows widefield microscopy pictures of U-2OS and HTB-88 cells treated with 2-OH BNPP1 drug. The lower confluence of cells is evident in the presence of the inhibitor, which is clearly at 20 μM. To confirm this observation, we performed colony formation assays (Figure 4G), which showed a statistically significant inhibition in the number of formed colonies for both cell lines principally treated at 20 μM, with the inhibitor compared to the colony number formed for non-treated or treated with DMSO (vehicle) cells. Furthermore, proliferation assays showed that 54% of U-2OS and 50% of HTB-88 cells diminished the proliferation rate in the presence of the BUB1 activity inhibitor at this concentration (Figure 4H). These results corroborate that the inhibition of BUB1 kinase precludes cell proliferation of the sarcoma-derived cell lines.

### 3.5. The Overall Survival of Sarcoma Patients Is Predicted by BUB1 Expression

**Figure 5:**
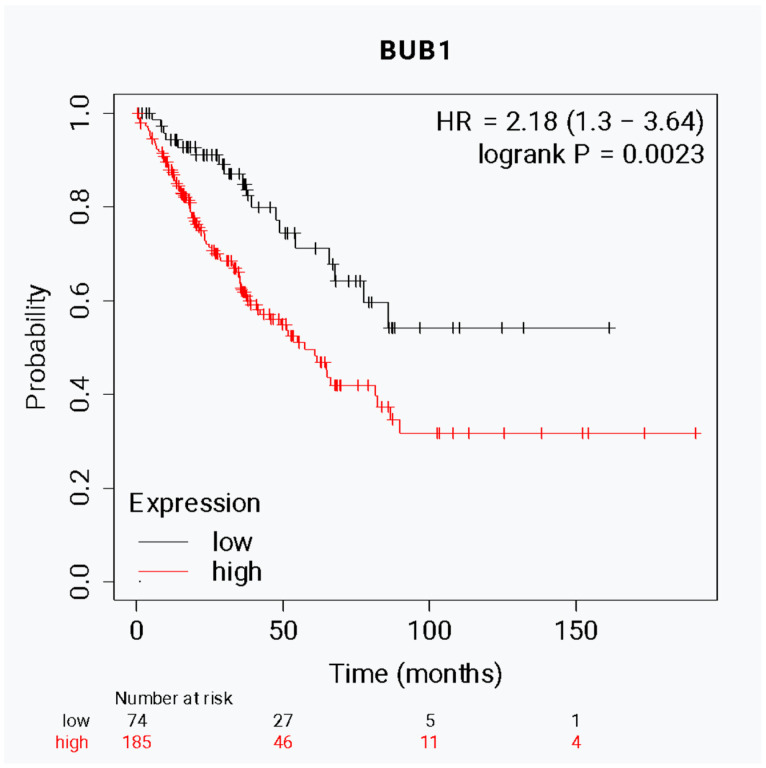
Expression levels of BUB1 are associated with overall survival in sarcoma patients. Kapplan–Meier analysis correlates the BUB1 levels (high or low) in tissue samples (*n* = 259) of sarcomas with overall survival. Hazar Ratio = 2.18 (1.3–3.64). *p* = 0.0023.

Finally, to investigate the potential role of BUB1 expression as a prognostic marker in sarcoma patients, the expression data from 259 sarcoma tissues were obtained from TCGA, and the median of BUB1 expression was determined and subdivided into two groups: (1) high expression and (2) low expression. The survival analysis was performed considering these levels of expression. The high expression of this kinase was associated with a median OS of 21.6 months, while the low expression was associated with a median OS of 47.87 months (*p* = 0.0023; HR: 2.18; IC95%: 1.3–3.64) (Figure 5). This data supports the main role of BUB1 in sarcoma progression and allows us to propose this kinase as a promising therapeutic target, potentially improving treatment strategies and patient outcomes by limiting tumor growth.

## 4. Discussion

Sarcomas are mesenchymal tumors developed in soft or bone tissues with different clinical, histological, biological, and molecular features. Around 70 subtypes of sarcomas have been reported, which makes it difficult to establish a specific therapy for each one of them [37]. This heterogeneity has resulted in the development of therapeutic strategies for these tumors based on general utility without taking into account their particularities. Sarcomas treatment includes surgery, chemotherapy, and radiotherapy. For metastatic disease, chemotherapy is the gold standard, where alkylating agents such as doxorubicin or ifosfamide are the first line of selected drugs [38,39]. A serious problem of chemotherapy is its high toxicity and low efficacy, which lead to poor clinical outcomes for sarcoma patients [16]. Recently, targeted therapies focused on molecular features were implemented in clinical practice, particularly for gastrointestinal stromal tumors (GISTs), characterized by the hyperactivity of c-KIT and PDFRα receptors [40]. Tyrosine kinase inhibitors such as imatinib, sunitinib, regorafenib, and repritinib are the gold standard treatment to block the kinase activity of these receptors. Another multitargeted TKI approved by the FDA for some metastatic soft tissue sarcomas treatment is Pazopanib [41]. However, in a phase II clinical trial, this drug had no shown impact on the growth of adipocytic sarcomas [42], demonstrating the high heterogeneity of RTK activity between subtypes of sarcoma tumors and encouraging the design of studies that consider a wider molecular landscape.

This work aimed to identify molecular features shared between the fourth most frequent type of sarcomas. We focused on overexpressed kinases due to their well-established pivotal role in driving cancer progression. The overexpressed kinases in osteosarcoma, liposarcoma, and leiomyosarcoma are mainly serine/threonine kinases (BUB1, CKD4, HUNK, LKKK1, NEK2, PBK, PLK1, and PLK4), whereas TTK has a dual role (Tyrosine and serine/threonine kinase) and only MELK presents tyrosine phosphorylation activity. This is in contrast with a previous work in which it was demonstrated that the phosphorylation in sarcoma cell lines and tumors are mainly drivers for tyrosine kinases [43]. However, they have technical limitations, such as the usage of an MS phosphotyrosine data set, which lacks a comprehensive approach to all kinases. An advantage of our study is that it includes a whole gene expression profile that involves tyrosine and serine/threonine kinase profiles. Interestingly, when we determined the overexpressed kinases shared between the fourth sarcoma subtypes analyzed, only the Budding Uninhibited by Benzimidazoles 1 (BUB1) kinase consistently exhibited high expression levels. This serine/threonine kinase was first reported as essential for mitosis progression because it is part of the mitotic checkpoint complex (MCC) [44]. Later, the BUB1’s role in the spindle assembly checkpoint (SAC), in the maintaining of genome stability, in chromatid cohesion protection, telomere replication, and in DNA repair mechanisms activation was widely described [30]. Moreover, high expression of BUB1 in colon, pancreatic, lung, gastric cancer, and other solid tumors was reported previously [45,46,47]. These levels were associated with cell proliferation increase, drug resistance, and cancer progression. Our interactome assay reveals that BUB1 could be regulated by transcription factors and molecules, such as non-coding RNAs, all involved in cancer development. This evidence and the data showing high gene expression and protein levels in cell lines allow us to hypothesize that this kinase also has a central role in sarcomas. It has been known that BUB1 activity inhibition reduces the phosphorylation of ser473-AKT and Thr120-H2A proteins [31,48]. Specifically, it was demonstrated that BUB1 inhibition regulates AKT phosphorylation to modulate mTOR and ERK signaling pathways in lung carcinoma and osteosarcoma, respectively [19,20]. We validated it using the drug 20H-BNPP1 in osteosarcoma and leiomyosarcoma cell lines. The effect of this drug on sarcoma cells was reflected by the lower phosphorylation rates of AKT and H2A proteins and cell proliferation inhibition, even though total protein levels did not change. Recently, BUB1 overexpression in osteosarcoma was demonstrated using cell lines and tissue samples. BUB1 inhibition reduces tumor growth and cell proliferation through PI3K/AKT signaling pathway inhibition [20]. Moreover, Sun and Cols reported that in this type of sarcoma high levels of BUB1 could be regulated by the family of homology 60A (FAM60A) protein [49]. BUB1 activity is essential for the spindle assembly checkpoint (SAC) during the M phase to regulate that DNA is distributed equally into daughter cells [50]. In addition, cancer cells display higher SAC dependence compared to normal cells; for this reason, the SAC inactivation by microtubule-disrupted agents favors mitosis arrest [19]. Recently, it was demonstrated that the inhibition of BUB1 activity sensitized cancer cells to the effect of docetaxel and paclitaxel in vitro and in vivo models. Both drugs belong to taxane agents that induce microtubule disruption to preclude mitosis progression and tumor growth [51,52]. BUB1 inhibition on small-cell lung cancer cells impacted cell growth, whereas this effect was not observed in normal epithelial cells, suggesting that these effects depend on the kinase levels [19]. Our observations about the proliferation effect in osteosarcoma and leiomyosarcoma cells by BUB1 activity inhibition allow us to propose this pharmacological intervention (mostly in combination with conventional therapeutic drugs for sarcomas as taxanes) not only for these two sarcomas but also for other types. Finally, the clinical utility of BUB1 kinase expression in sarcomas was evident. High expression levels of the kinase were associated with poor overall survival of patients. This observation correlates with the results previously described in osteosarcoma, fibrosarcoma, histiocytoma, and epithelial tumors [20,53,54]. In addition, BUB1 expression was proposed as a predictor of response to immunotherapy in breast cancer tumors [55]. Determining the association between BUB1 levels and other clinicopathological features in sarcomas remains as a perspective.

## 5. Conclusions

This study highlights the overexpression of effector molecules such as kinases in the four most frequent types of sarcomas. Our analysis allowed us to identify a molecule that could clearly be a target of pharmacological inhibition in the treatment of both bone and soft tissue sarcomas. This strengthens the idea of the use of target therapies focused on the inhibition of BUB1. As perspectives of this work, the deep analysis of the molecular aspects that modulate the inhibition of this kinase and how it affects the biology of the assayed subtypes of sarcomas remains.

## Figures and Tables

**Table 1:** General information of GEO data collected.

Type of Sarcoma	GEO Accession	Sequencing Technology	Samples
Osteosarcoma	GSE218035	Expression profiling by high-throughput sequencing: Oxford Nanopore Technologies (ONT) long-read RNA-Seq	Tumor and adjacent normal tissues from 23 osteosarcoma patients paired with adjacent healthy tissue.
Liposarcoma	GSM6049698	Expression profiling by high-throughput sequencing: Illumina NovaSeq 6000	Expression data from 18 liposarcoma and 20 adjacent healthy tissue samples.
Synovial sarcoma	GSE144190	Expression profiling by high-throughput sequencing: Illumina NovaSeq 6000	Tumor and adjacent normal tissues from 10 synovial sarcoma patients paired with adjacent healthy tissue.
Leiomyosarcoma	GSE45510	Expression profiling by high-throughput sequencing: Illumina Genome Analyzer IIx	Expression data from 99 leiomyosarcoma tissue samples.
	GSE54511	Illumina Genome Analyzer IIx	Expression data of 4 healthy tissue samples from myometrium.

## Data Availability

Data is contained within the article or supplementary material

## Supplementary Materials

The following supporting information can be downloaded at: https://www.mdpi.com/article/10.3390/biom15071046/s1. Figure S1. Raw images of the western blot in Figure 4B; Figure S2. Raw images of the western blot in Figure 4D
