# Supplementary figures and images for: BUB1 an Overexpressed Kinase in Sarcoma: Finding New Target Therapy for Osteosarcoma, Liposarcoma, Synovial Sarcoma, and Leiomyosarcoma
Source: Biomolecules. 2025 Jul 18;15(7):1046. doi: 10.3390/biom15071046 (PMC12293735; doi:10.3390/biom15071046)

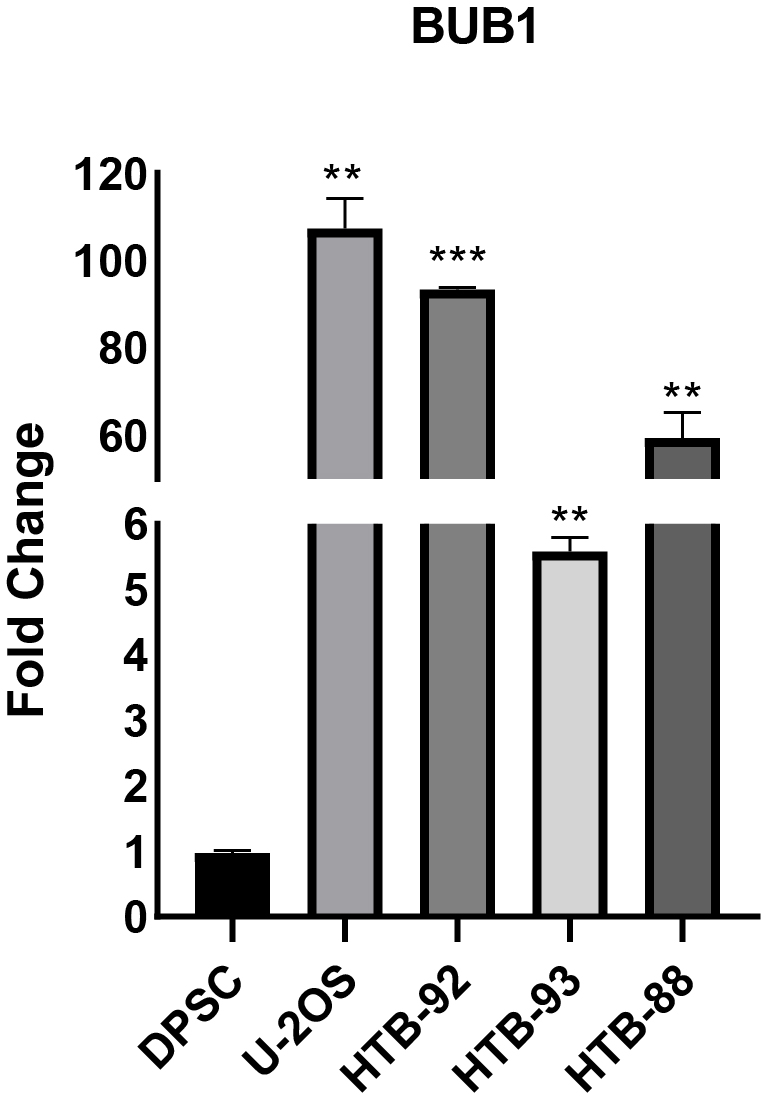

Supplement: Supplementary file 1 [file biomolecules-15-01046-s001.zip › Raw data fig.4 /FIGURE 4A/Grafica final BUB1.jpg]

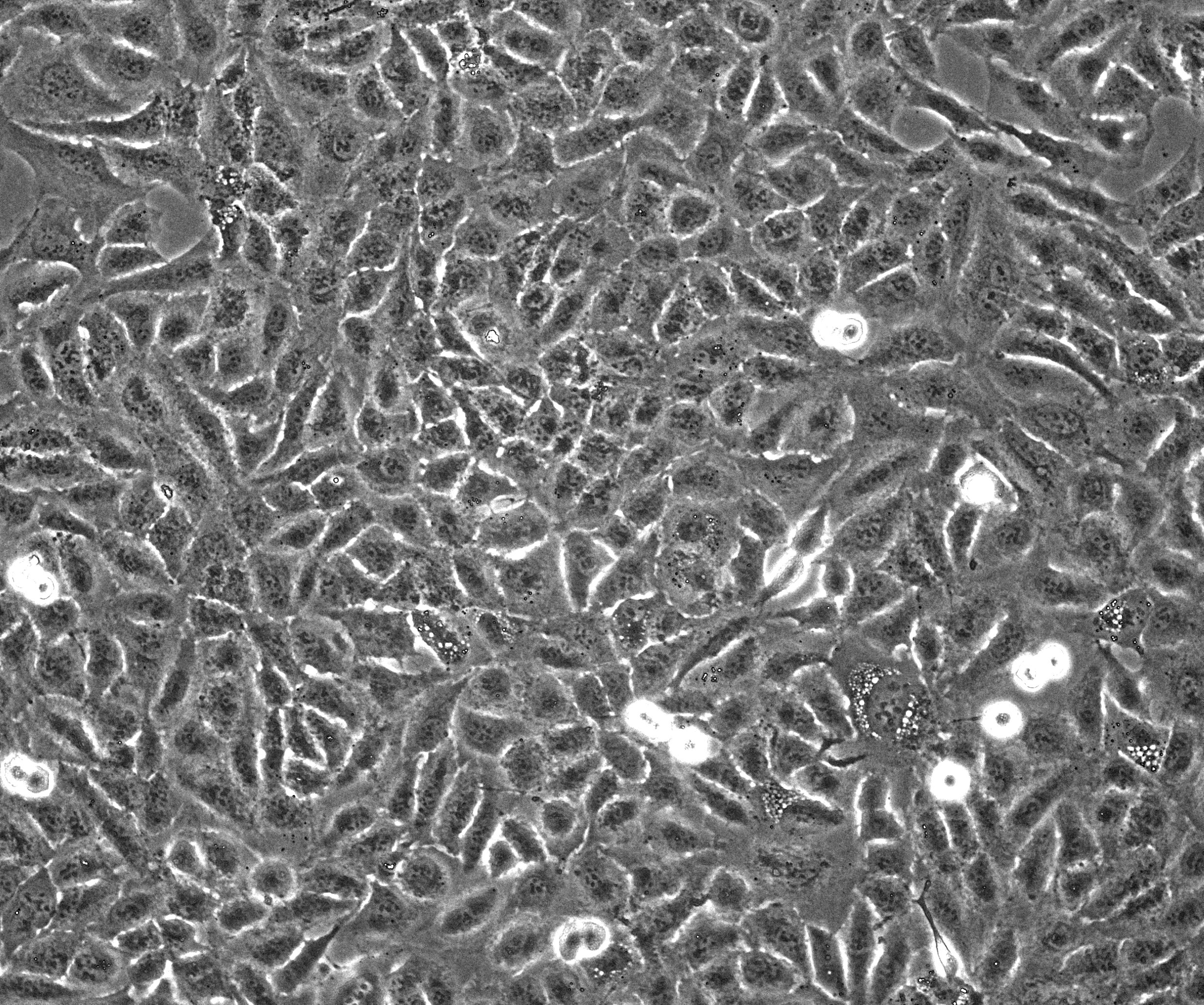

Supplement: Supplementary file 1 [file biomolecules-15-01046-s001.zip › Raw data fig.4 /FIGURE 4F/NT2 U2OS.jpg]

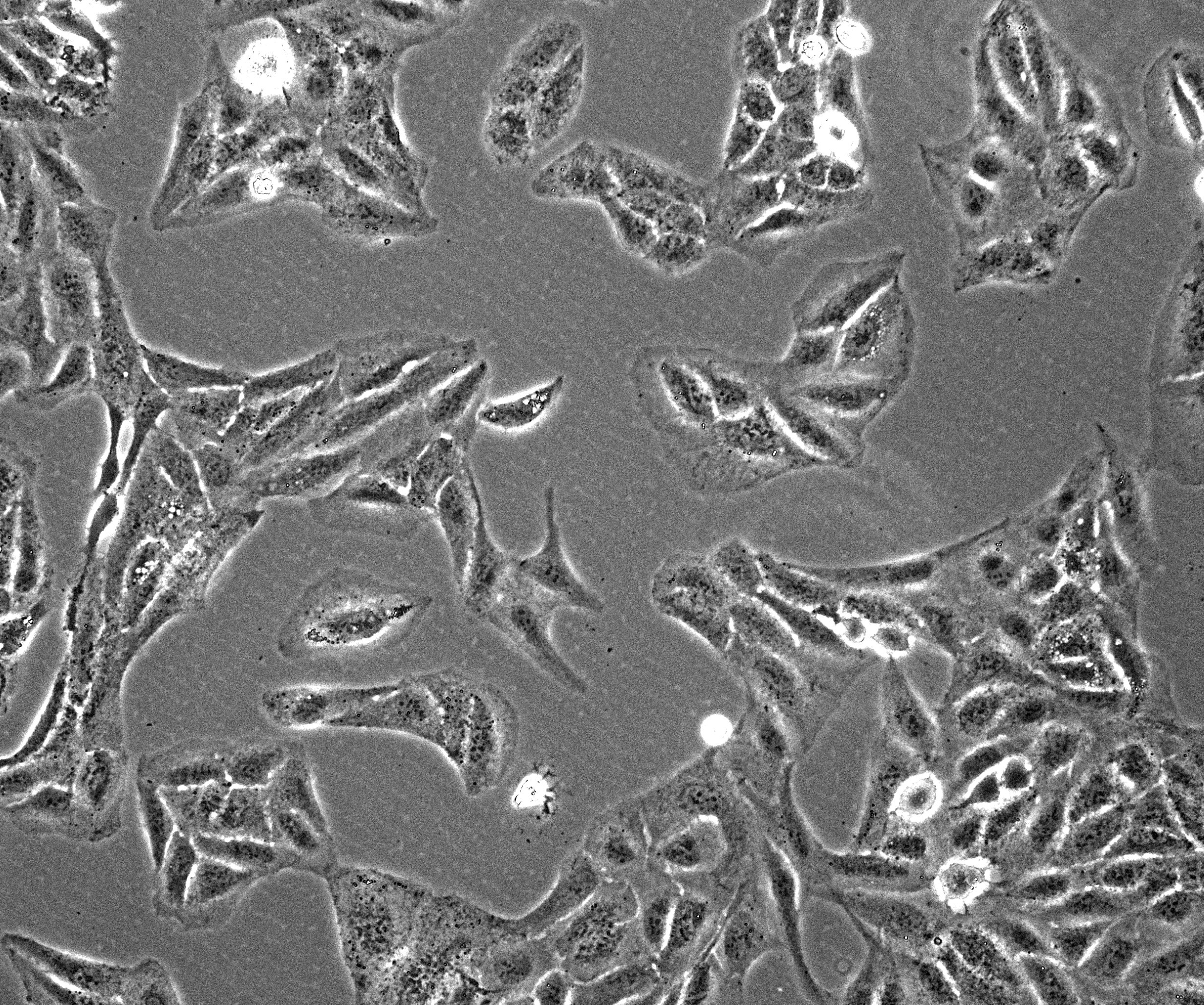

Supplement: Supplementary file 1 [file biomolecules-15-01046-s001.zip › Raw data fig.4 /FIGURE 4F/BNP 5microM 1 U2OS.jpg]

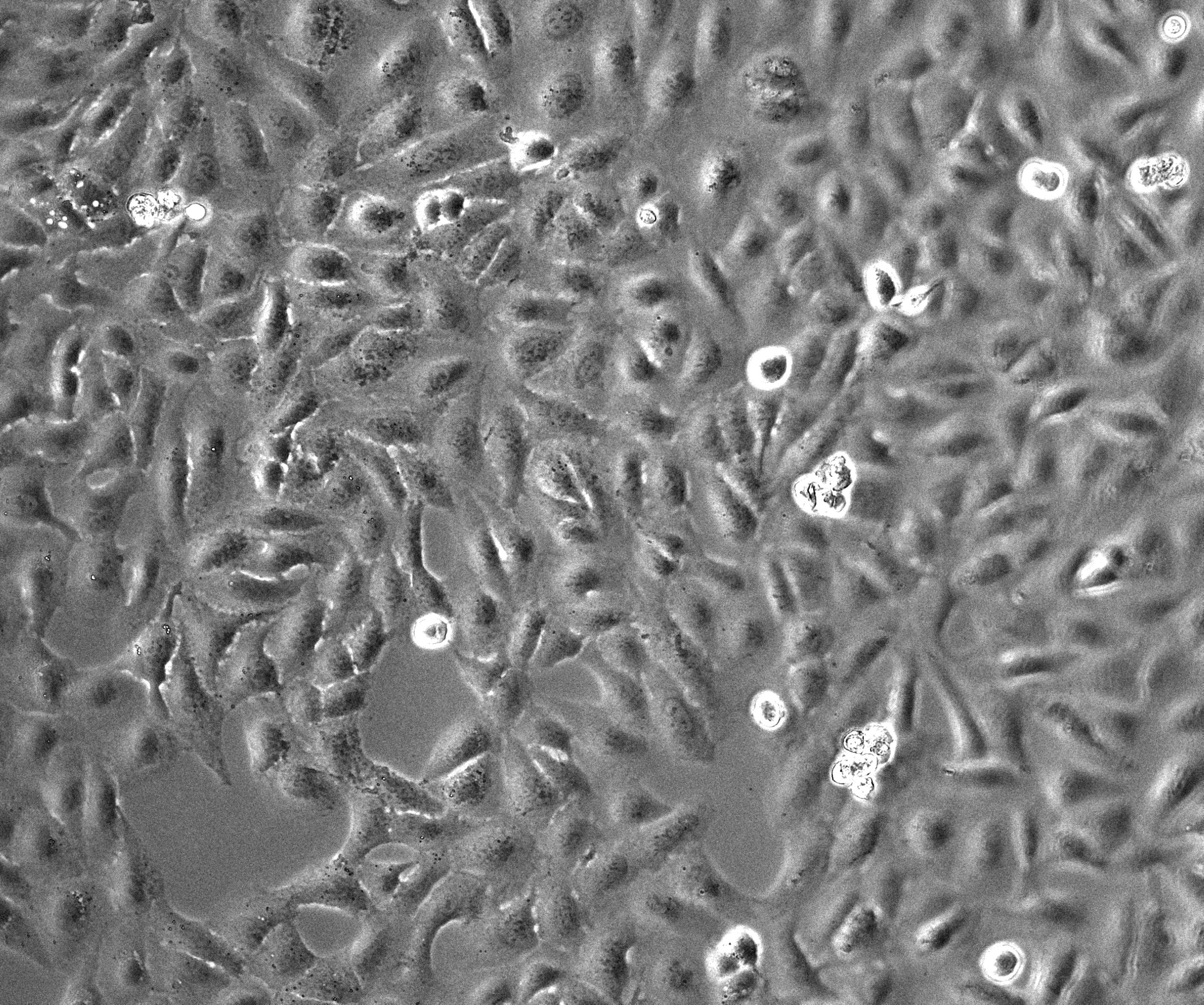

Supplement: Supplementary file 1 [file biomolecules-15-01046-s001.zip › Raw data fig.4 /FIGURE 4F/DMSO2 USO2.jpg]

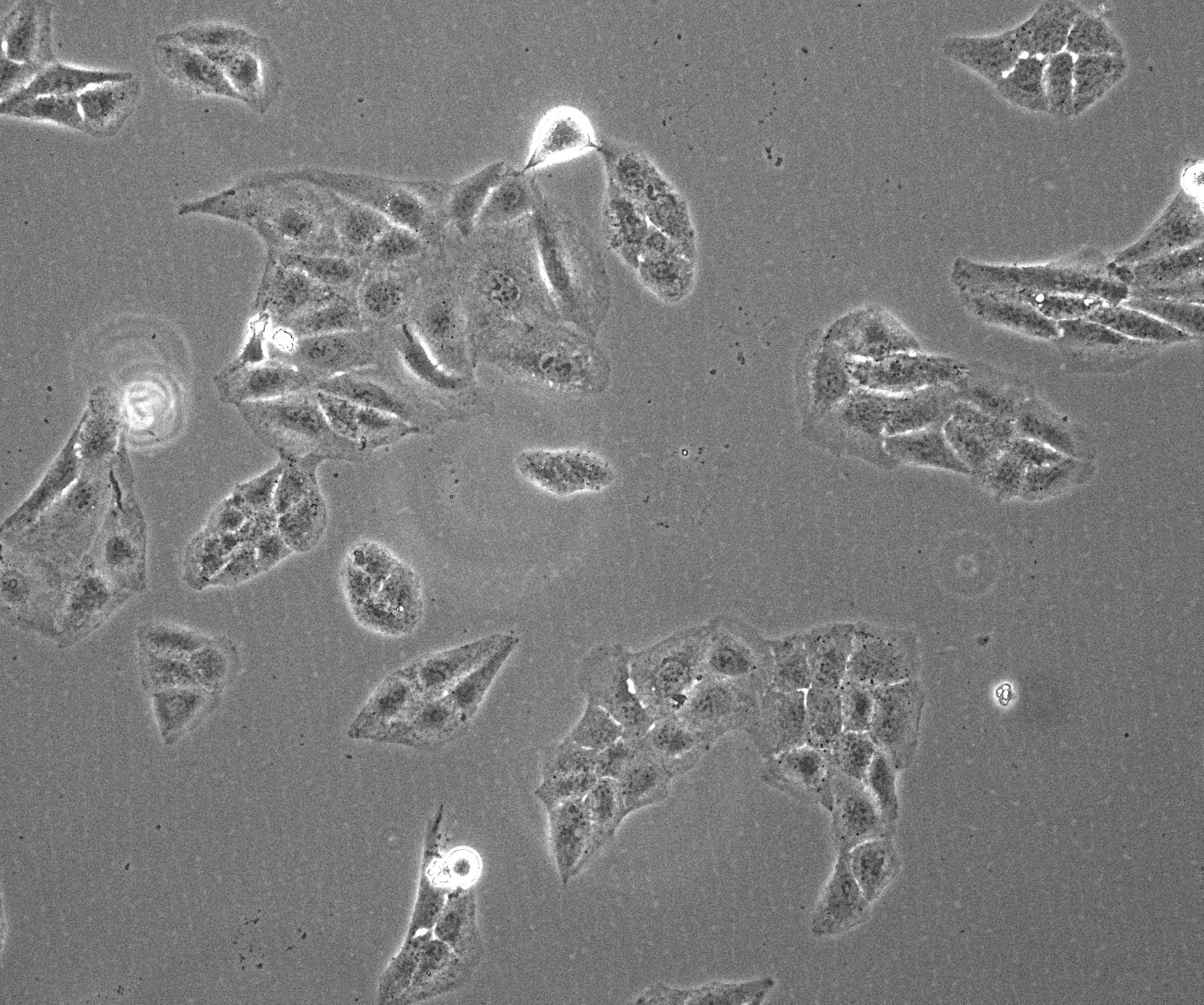

Supplement: Supplementary file 1 [file biomolecules-15-01046-s001.zip › Raw data fig.4 /FIGURE 4F/BNP 20microM 2U2OS.jpg]

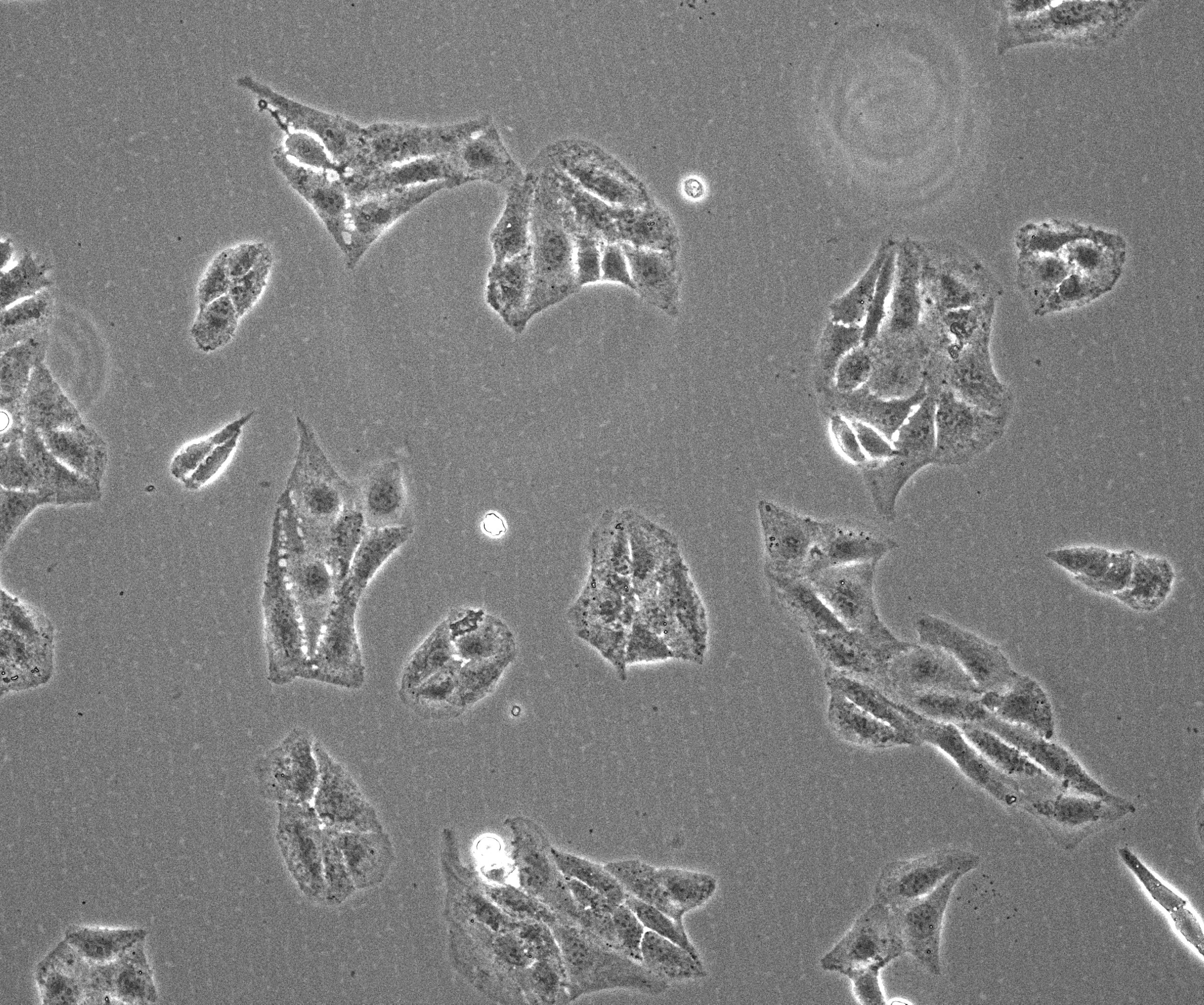

Supplement: Supplementary file 1 [file biomolecules-15-01046-s001.zip › Raw data fig.4 /FIGURE 4F/BNP 20microM 1 U2OS.jpg]

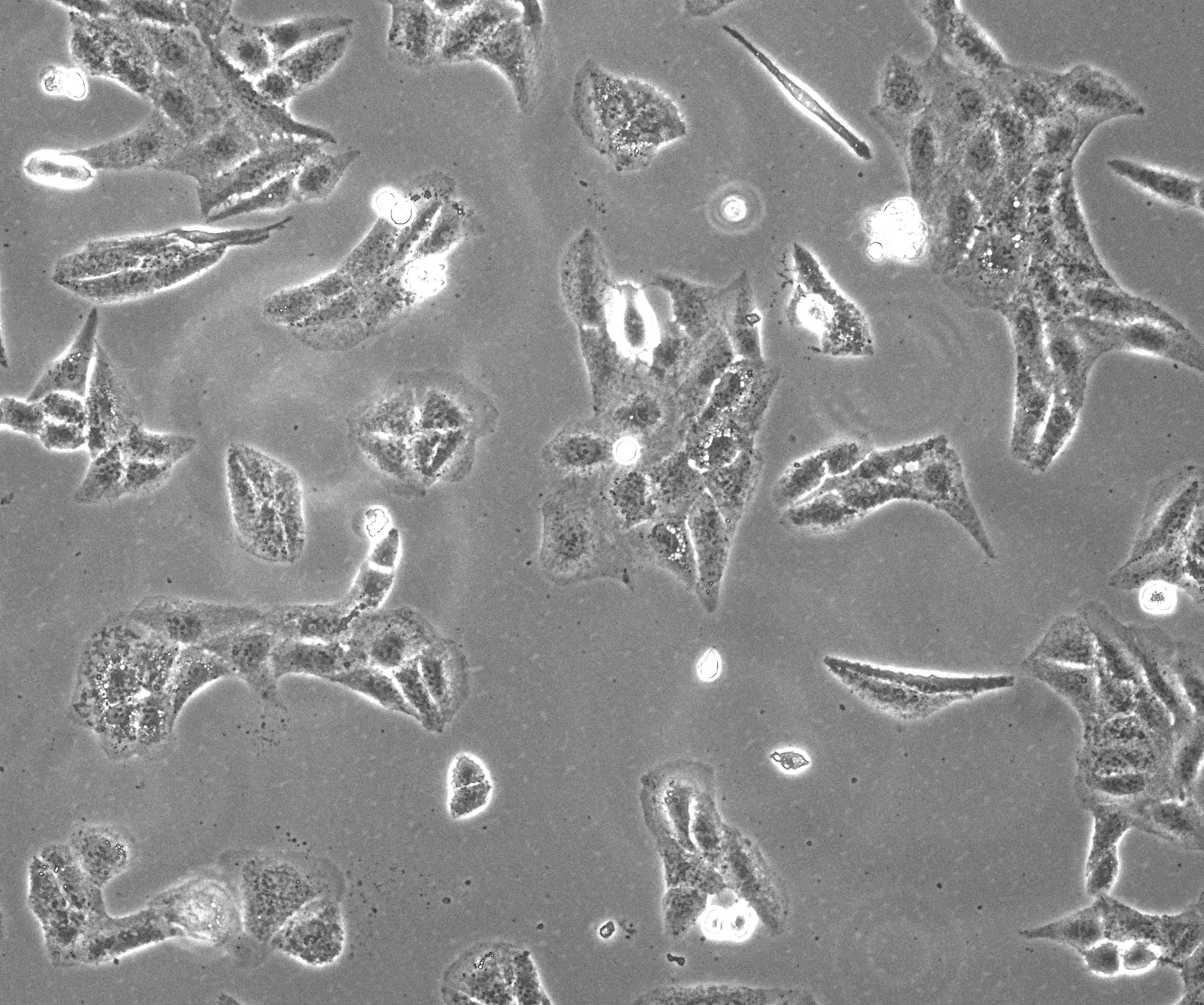

Supplement: Supplementary file 1 [file biomolecules-15-01046-s001.zip › Raw data fig.4 /FIGURE 4F/BNP 10microM 2U2OS.jpg]

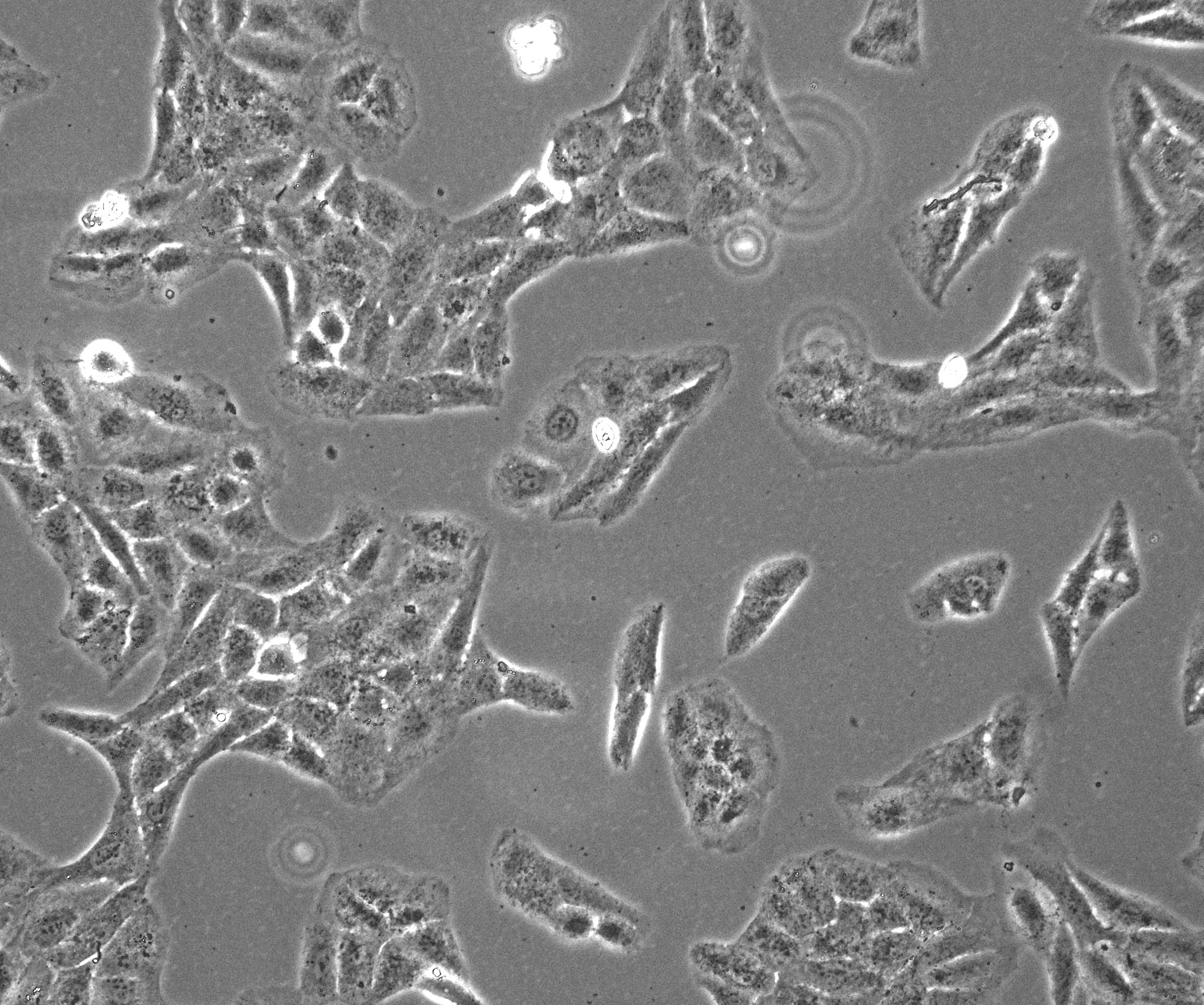

Supplement: Supplementary file 1 [file biomolecules-15-01046-s001.zip › Raw data fig.4 /FIGURE 4F/BNP 10microM 1 U2OS.jpg]

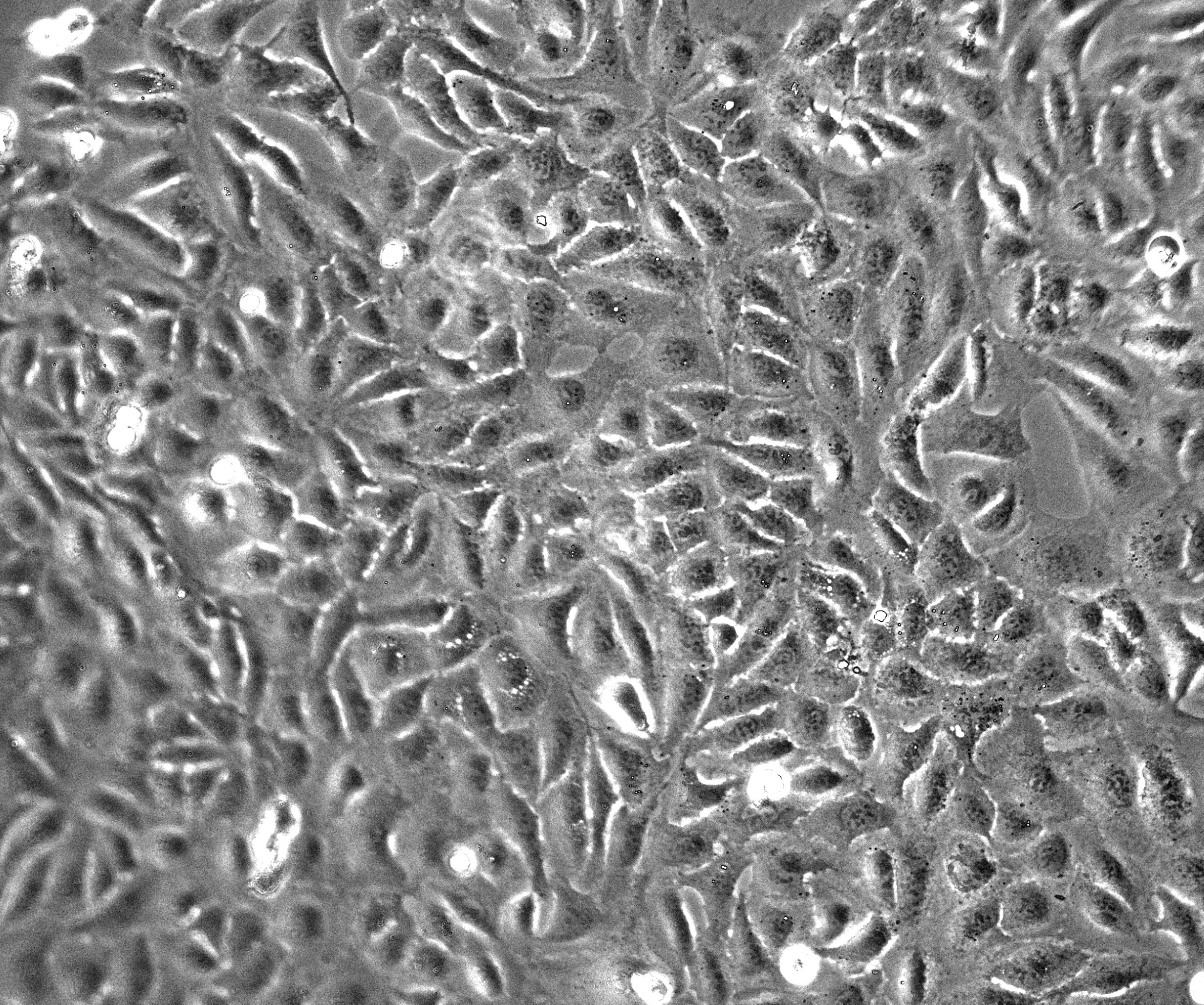

Supplement: Supplementary file 1 [file biomolecules-15-01046-s001.zip › Raw data fig.4 /FIGURE 4F/DMSO1 USO2.jpg]

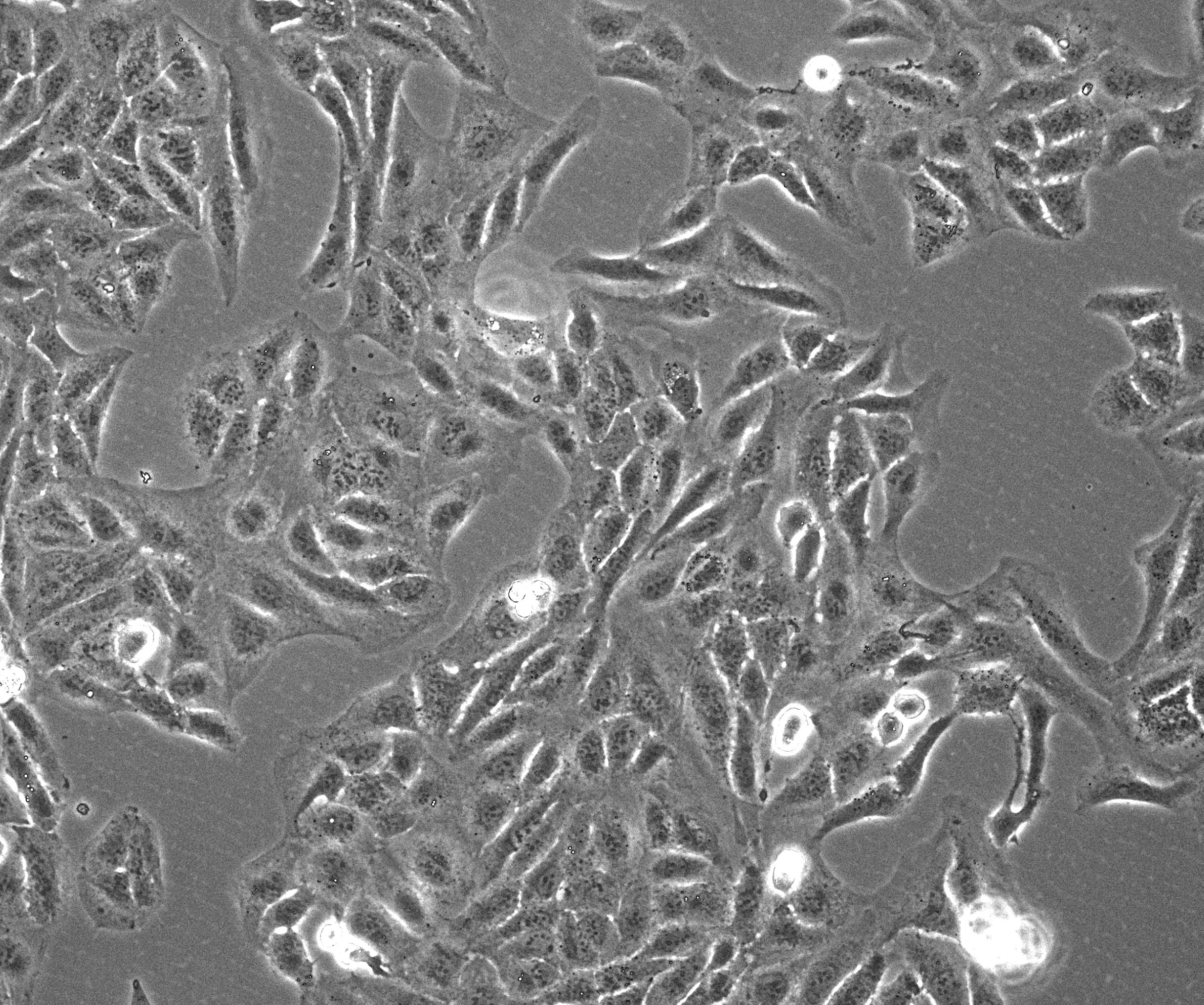

Supplement: Supplementary file 1 [file biomolecules-15-01046-s001.zip › Raw data fig.4 /FIGURE 4F/BNP 5microM 2U2OS.jpg]

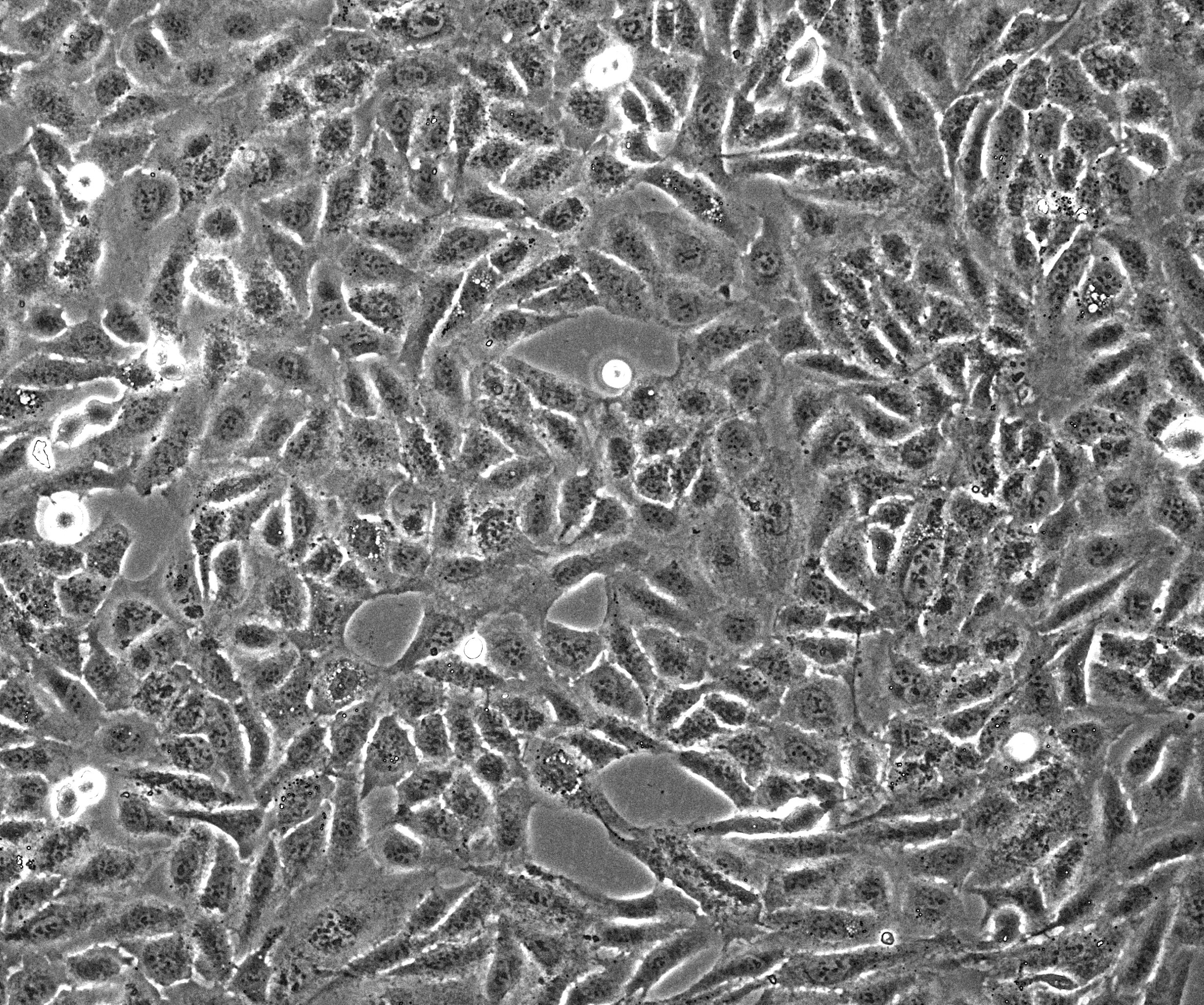

Supplement: Supplementary file 1 [file biomolecules-15-01046-s001.zip › Raw data fig.4 /FIGURE 4F/NT1 U2OS.jpg]

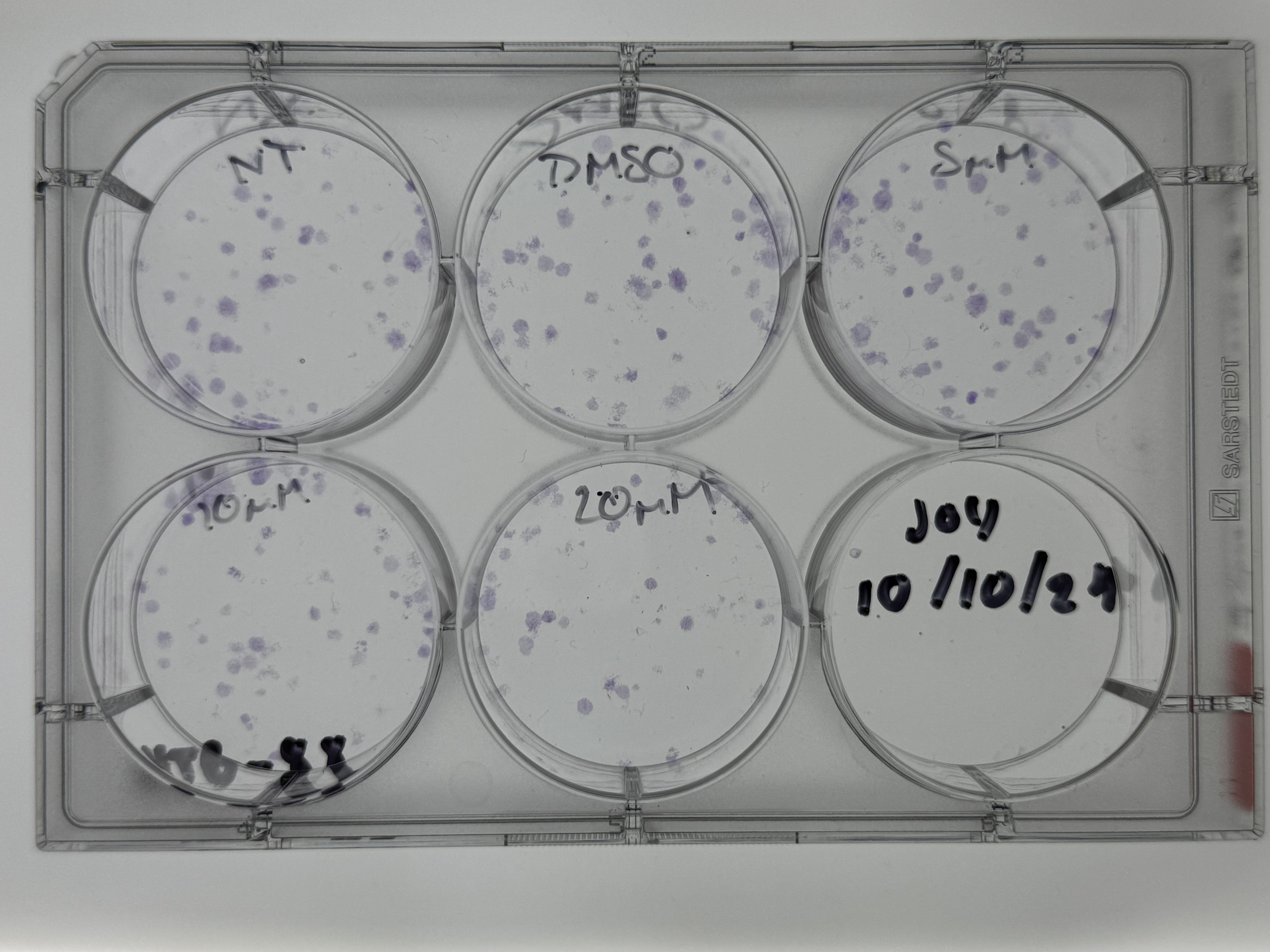

Supplement: Supplementary file 1 [file biomolecules-15-01046-s001.zip › Raw data fig.4 /FIGURE 4G/HTB-88 Colony Assay 2.JPEG]

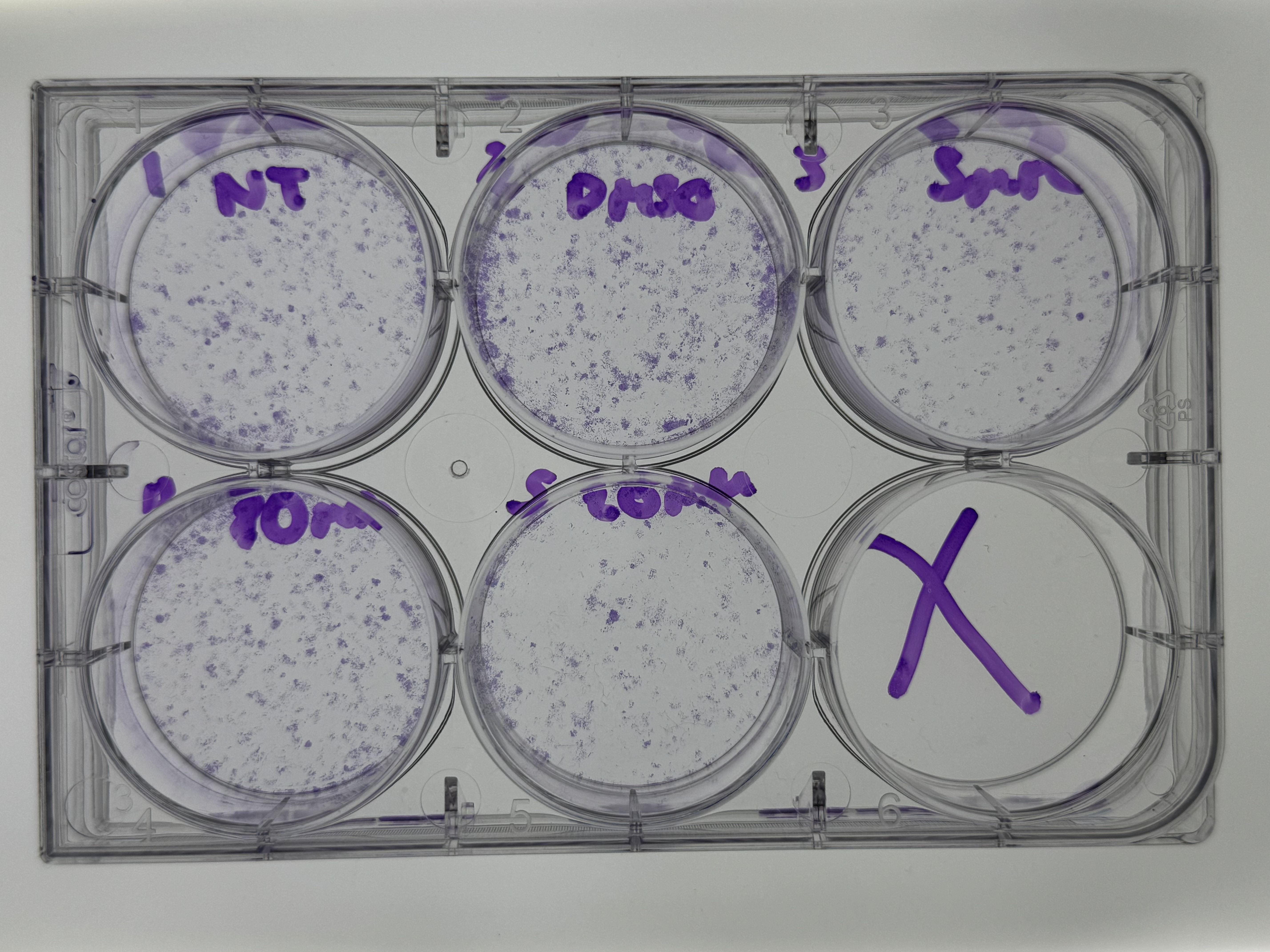

Supplement: Supplementary file 1 [file biomolecules-15-01046-s001.zip › Raw data fig.4 /FIGURE 4G/HTB-88 Colony Assay 3.JPEG]

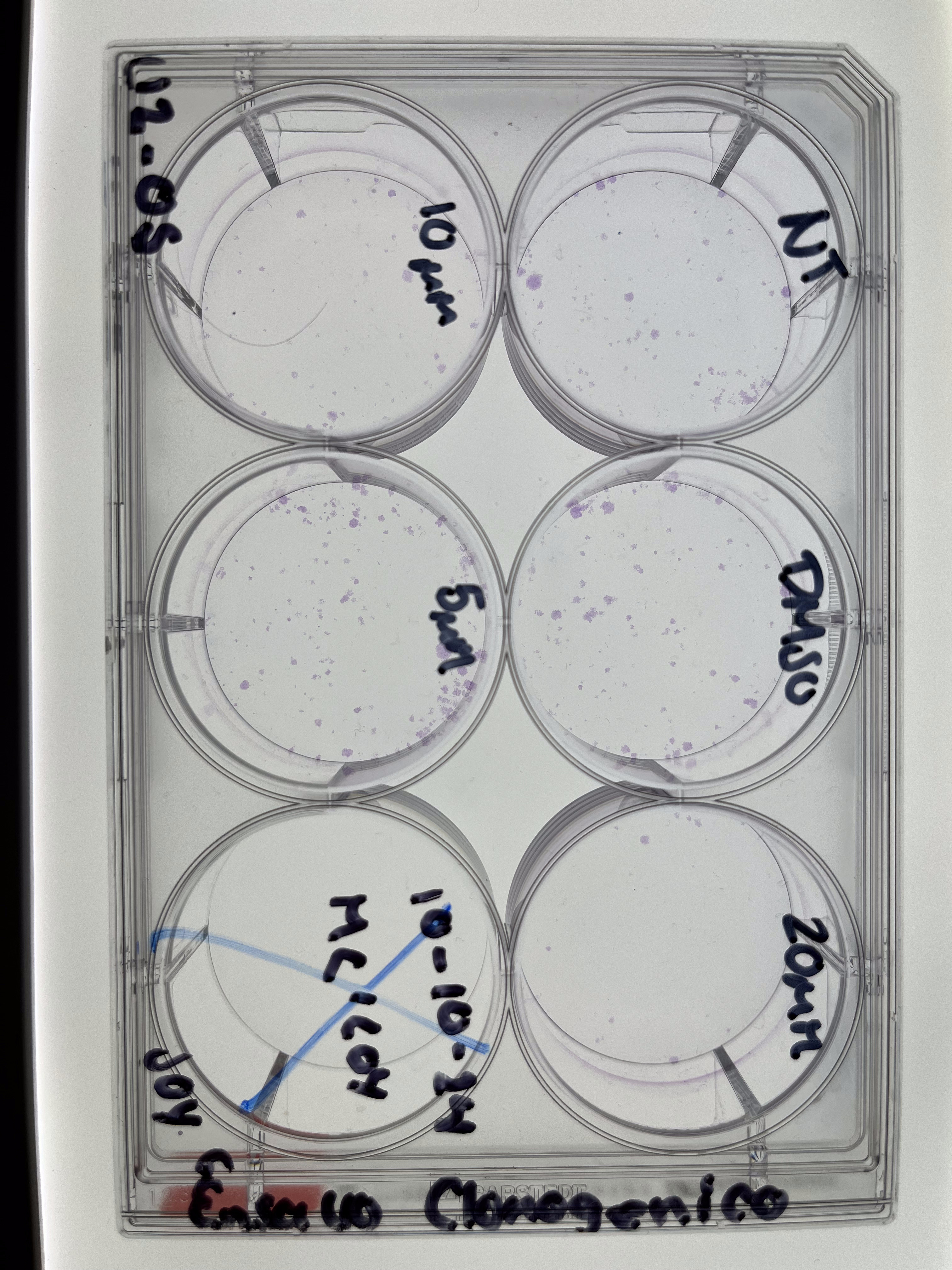

Supplement: Supplementary file 1 [file biomolecules-15-01046-s001.zip › Raw data fig.4 /FIGURE 4G/U-2OS Colony Assay 2.jpg]

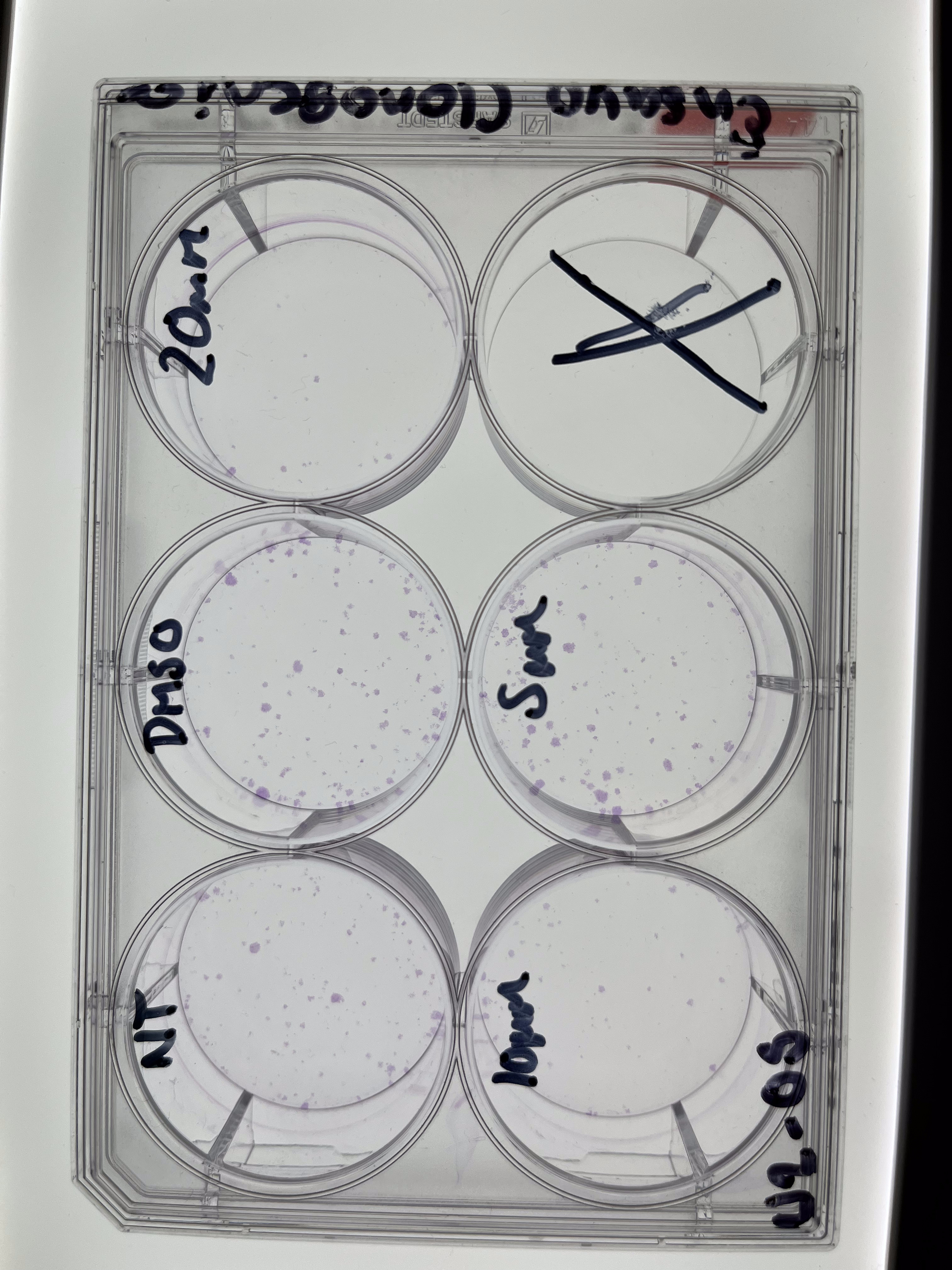

Supplement: Supplementary file 1 [file biomolecules-15-01046-s001.zip › Raw data fig.4 /FIGURE 4G/U-2OS Colony Assay 3.jpg]

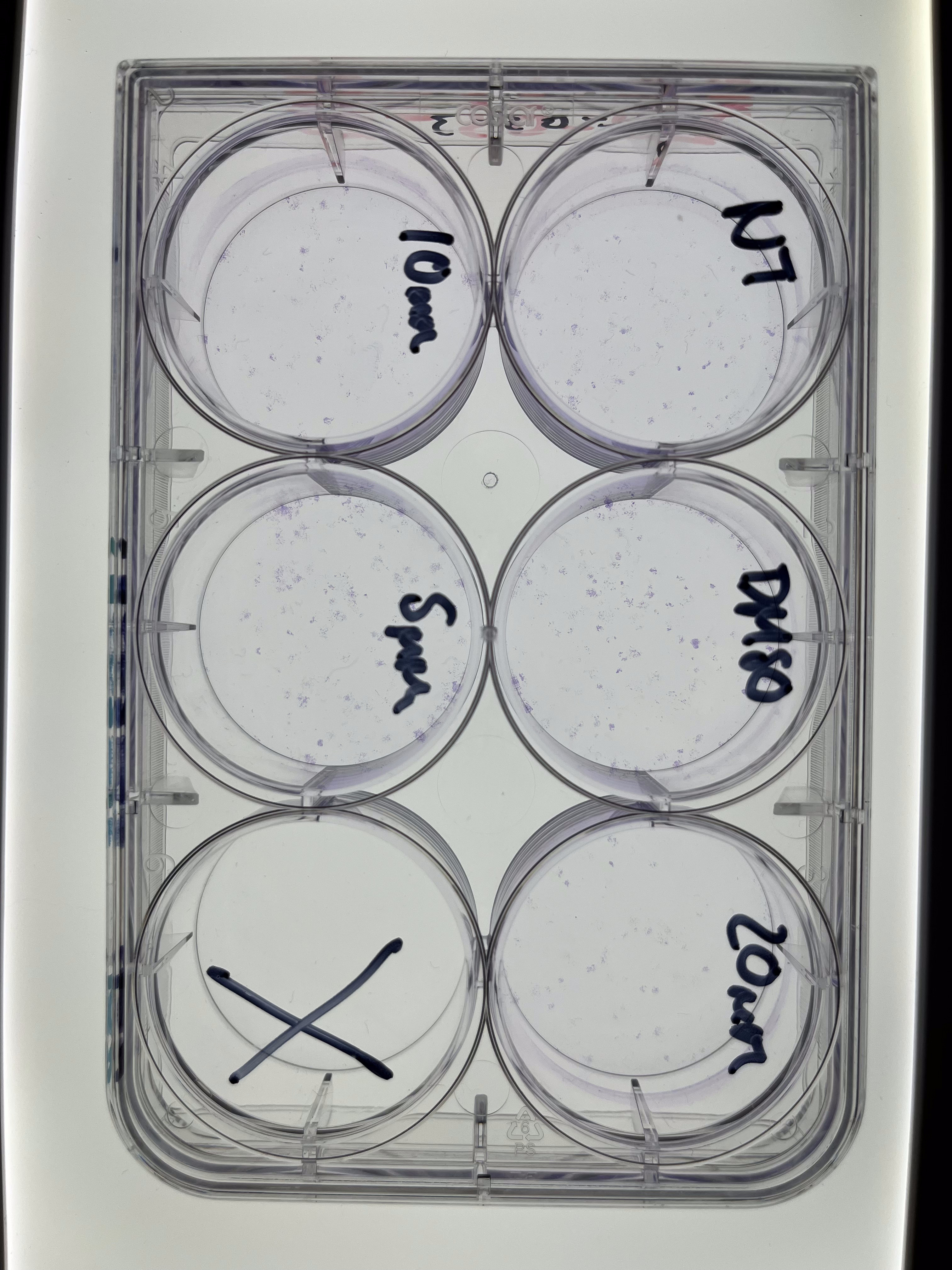

Supplement: Supplementary file 1 [file biomolecules-15-01046-s001.zip › Raw data fig.4 /FIGURE 4G/U-2OS Colony Assay 1.jpg]

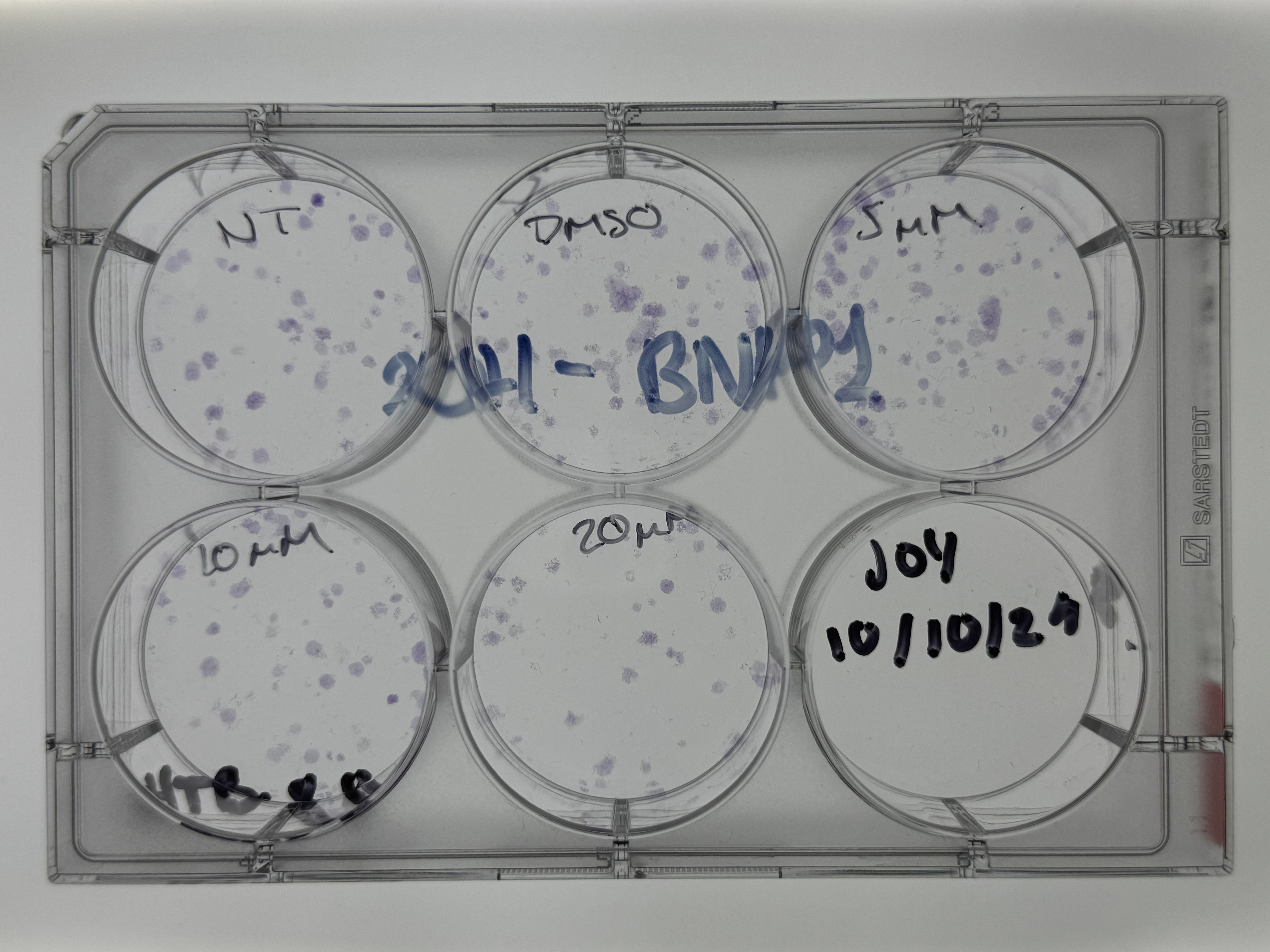

Supplement: Supplementary file 1 [file biomolecules-15-01046-s001.zip › Raw data fig.4 /FIGURE 4G/HTB-88 Colony Assay 1.JPEG]

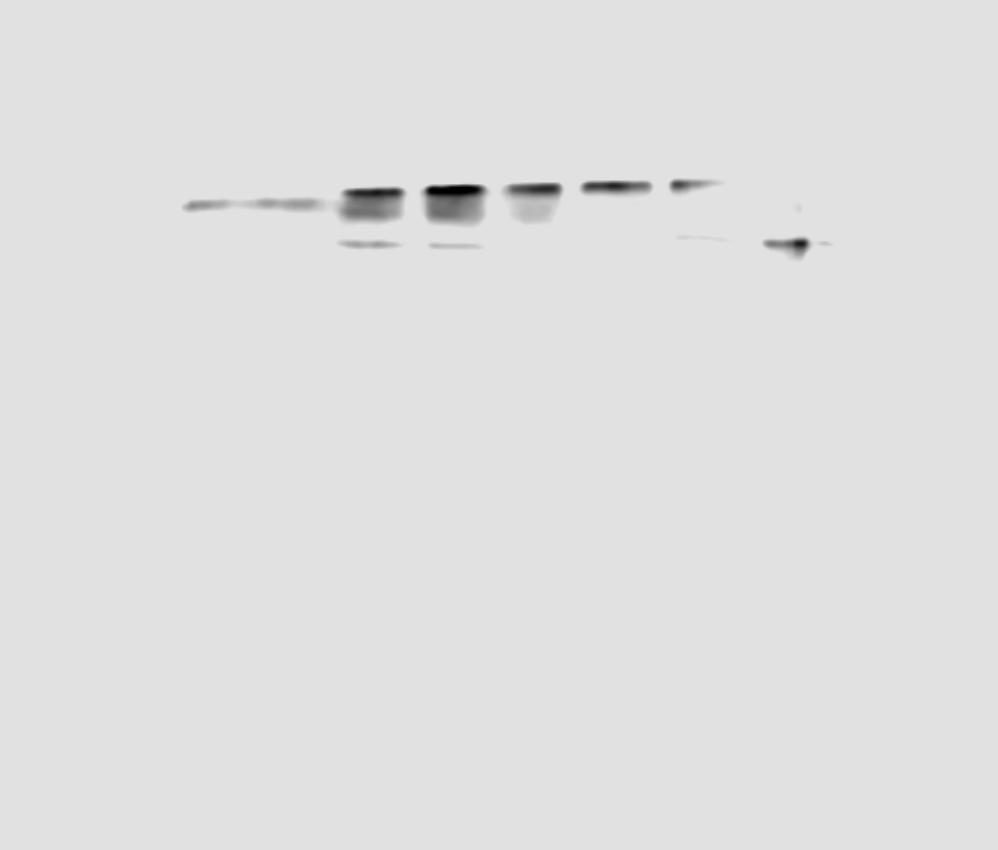

Supplement: Supplementary file 1 [file biomolecules-15-01046-s001.zip › Raw data fig.4 /FIGURE 4D/HTB-88/p-Thr120 H2A HTB-88.tif]

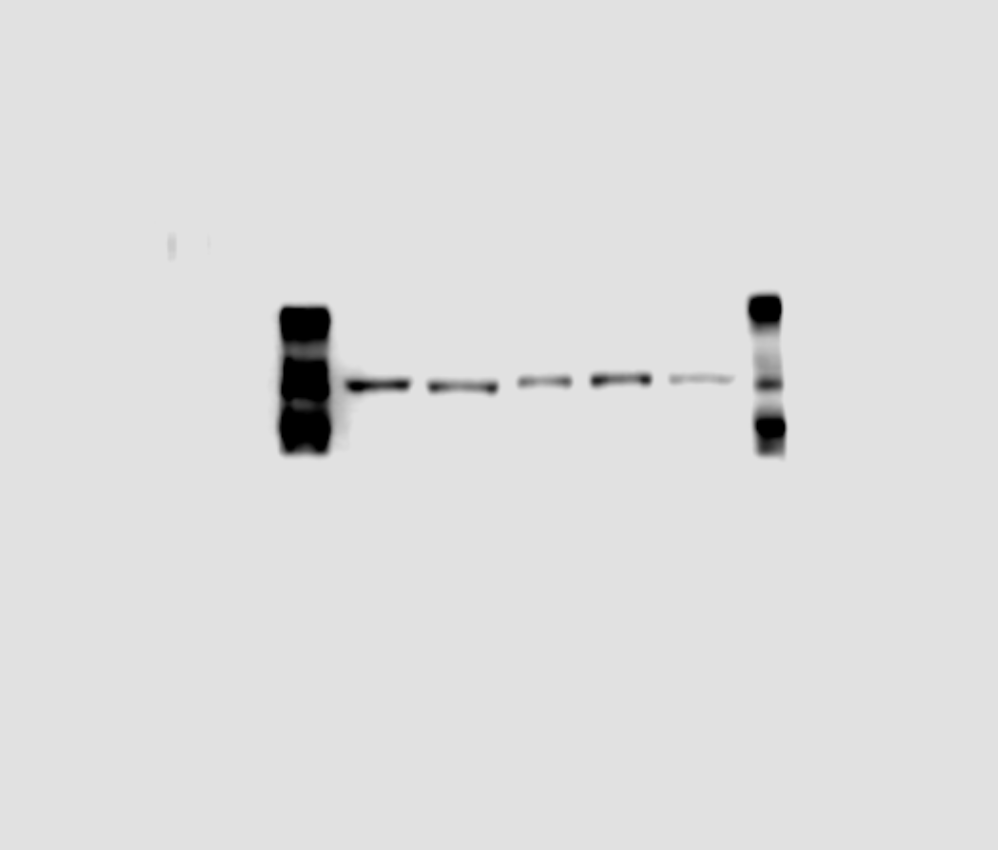

Supplement: Supplementary file 1 [file biomolecules-15-01046-s001.zip › Raw data fig.4 /FIGURE 4D/HTB-88/p-Ser473 AKT.tif]

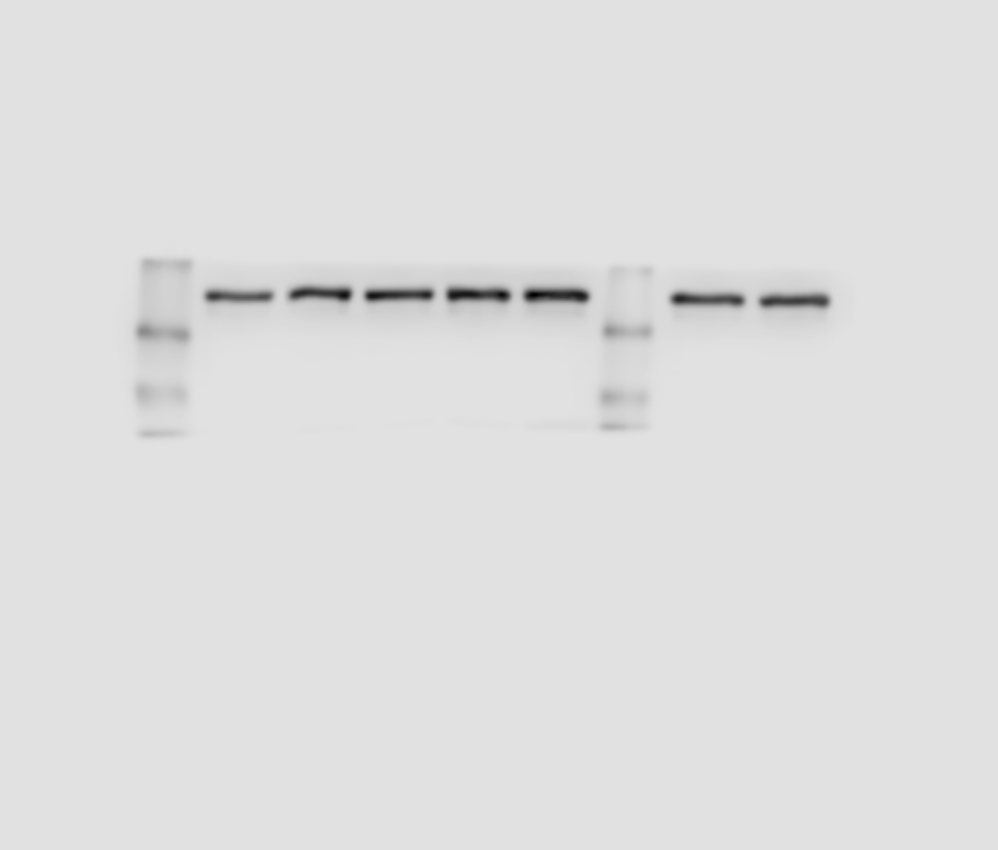

Supplement: Supplementary file 1 [file biomolecules-15-01046-s001.zip › Raw data fig.4 /FIGURE 4D/HTB-88/ ACTIN HTB-88.tif]

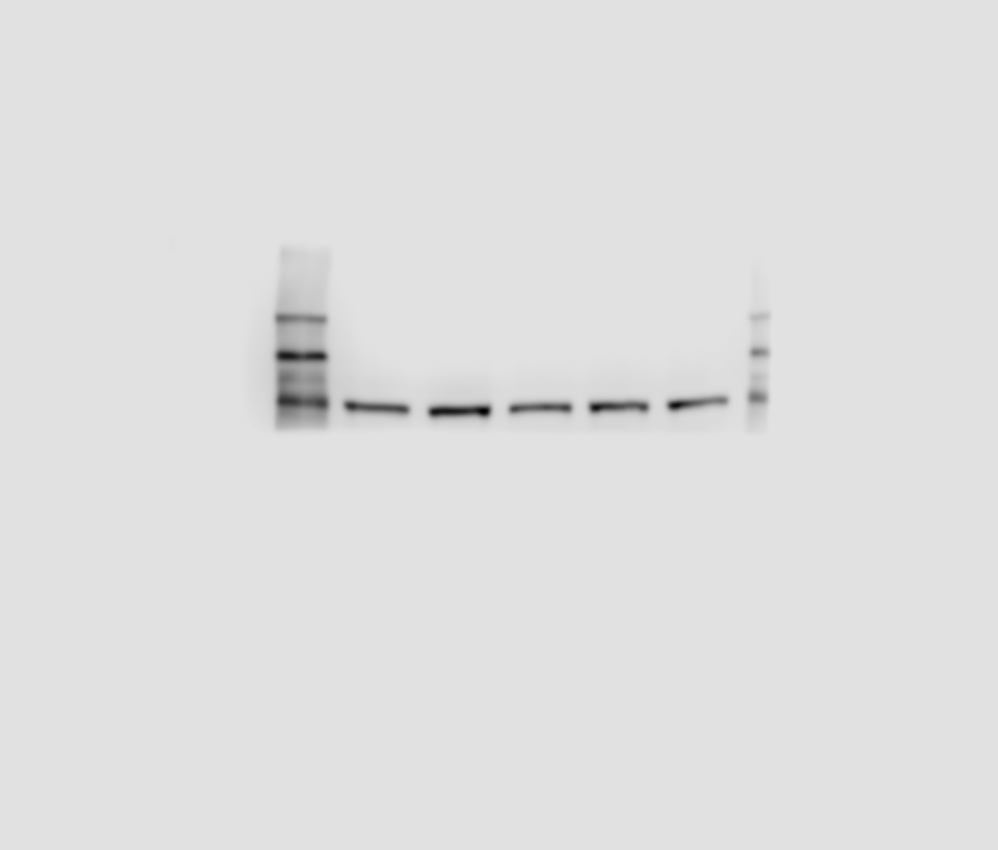

Supplement: Supplementary file 1 [file biomolecules-15-01046-s001.zip › Raw data fig.4 /FIGURE 4D/HTB-88/BUB1 TOTAL HTB-88.tif]

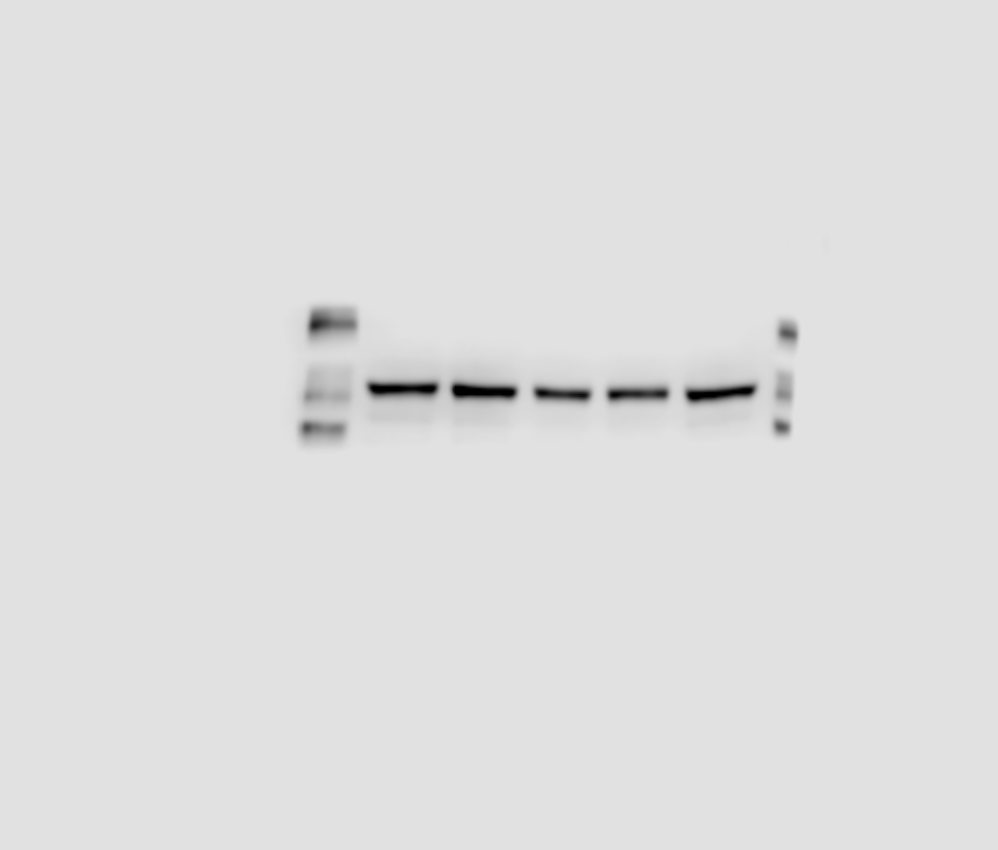

Supplement: Supplementary file 1 [file biomolecules-15-01046-s001.zip › Raw data fig.4 /FIGURE 4D/HTB-88/Total-AKT.tif]

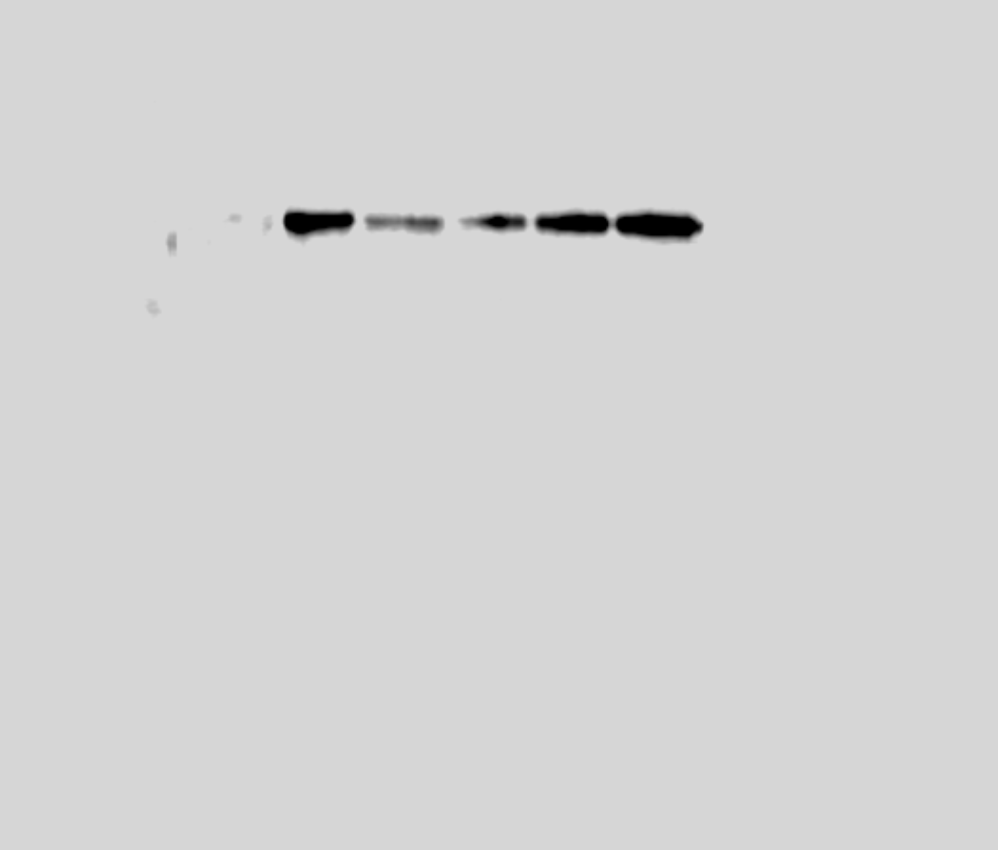

Supplement: Supplementary file 1 [file biomolecules-15-01046-s001.zip › Raw data fig.4 /FIGURE 4D/HTB-88/Total H2A HTB-88.tif]

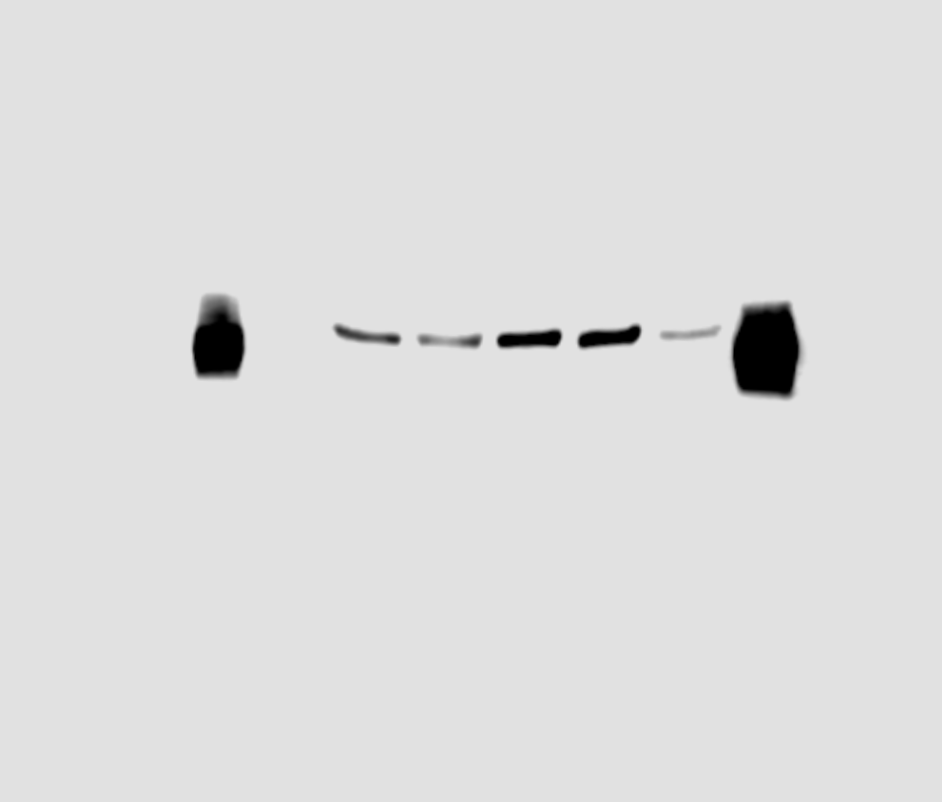

Supplement: Supplementary file 1 [file biomolecules-15-01046-s001.zip › Raw data fig.4 /FIGURE 4D/U-2OS/p-Ser473 AKT.tif]

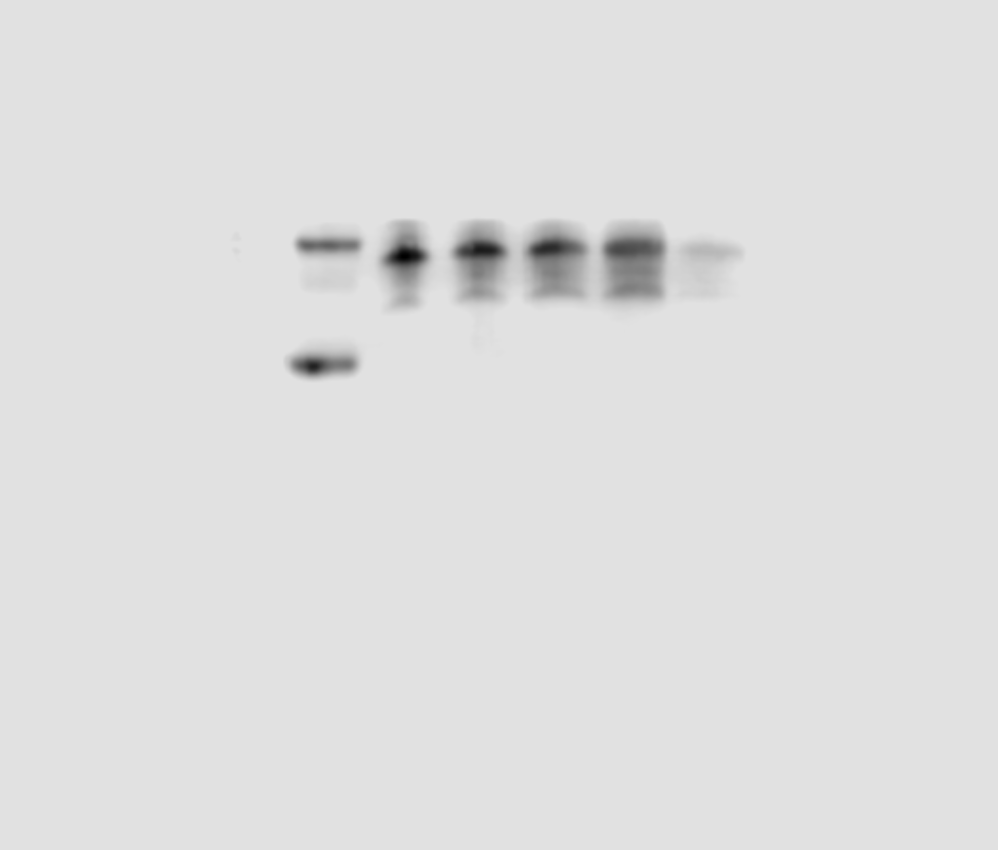

Supplement: Supplementary file 1 [file biomolecules-15-01046-s001.zip › Raw data fig.4 /FIGURE 4D/U-2OS/p-Thr120 H2A U-2OS.tif]

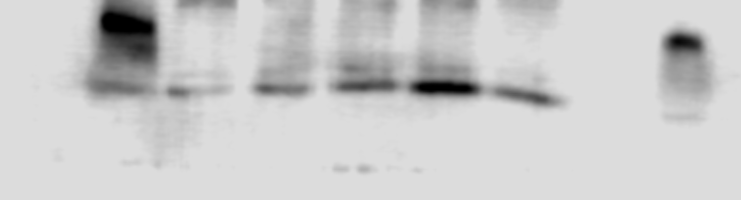

Supplement: Supplementary file 1 [file biomolecules-15-01046-s001.zip › Raw data fig.4 /FIGURE 4D/U-2OS/Total H2A U-2OS.tif]

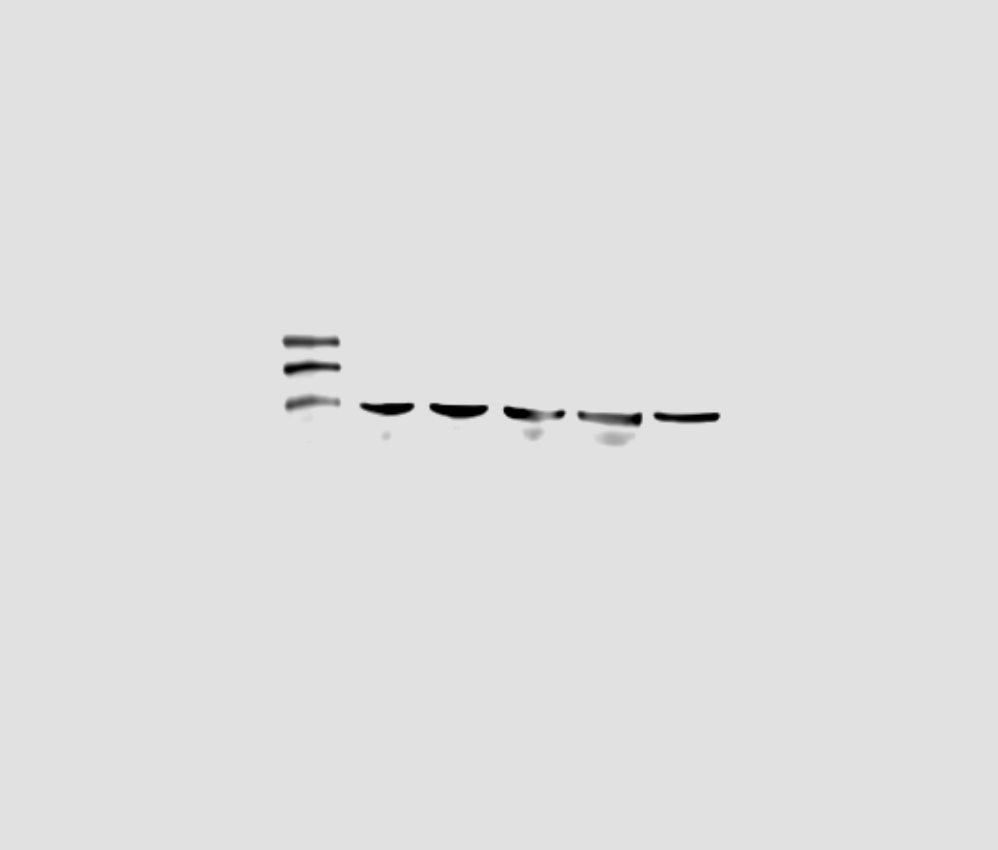

Supplement: Supplementary file 1 [file biomolecules-15-01046-s001.zip › Raw data fig.4 /FIGURE 4D/U-2OS/Total BUB1 U-2OS.tif]

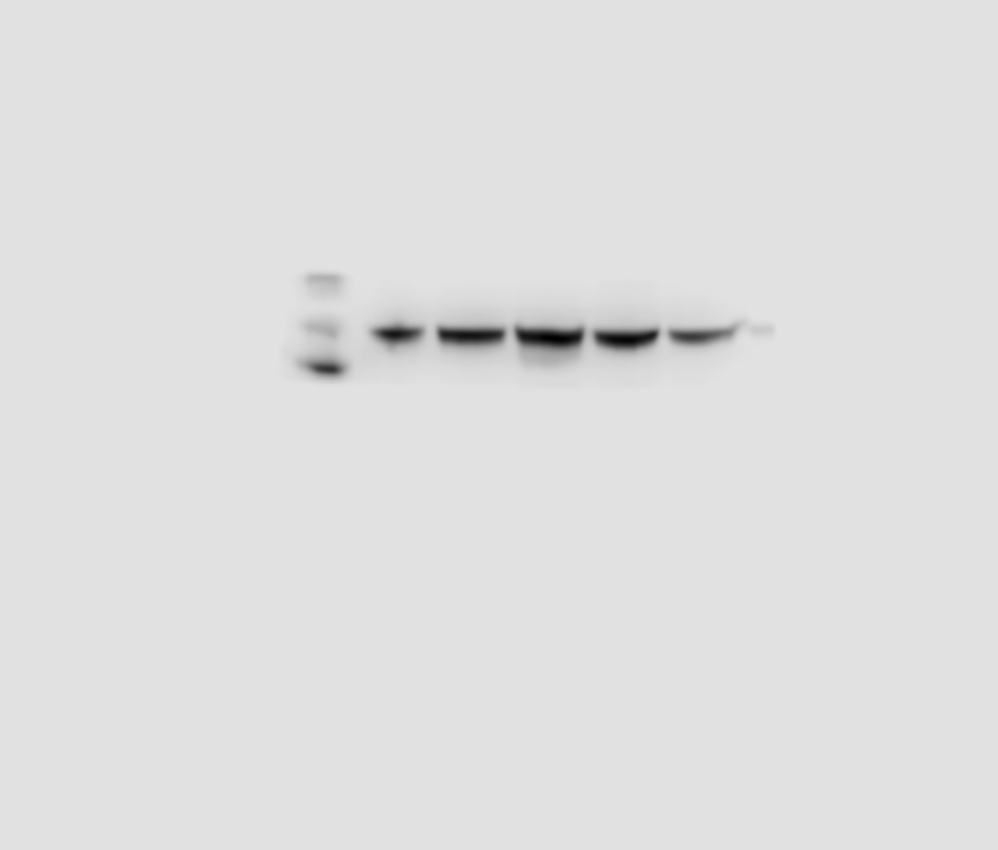

Supplement: Supplementary file 1 [file biomolecules-15-01046-s001.zip › Raw data fig.4 /FIGURE 4D/U-2OS/TOTAL AKT.tif]

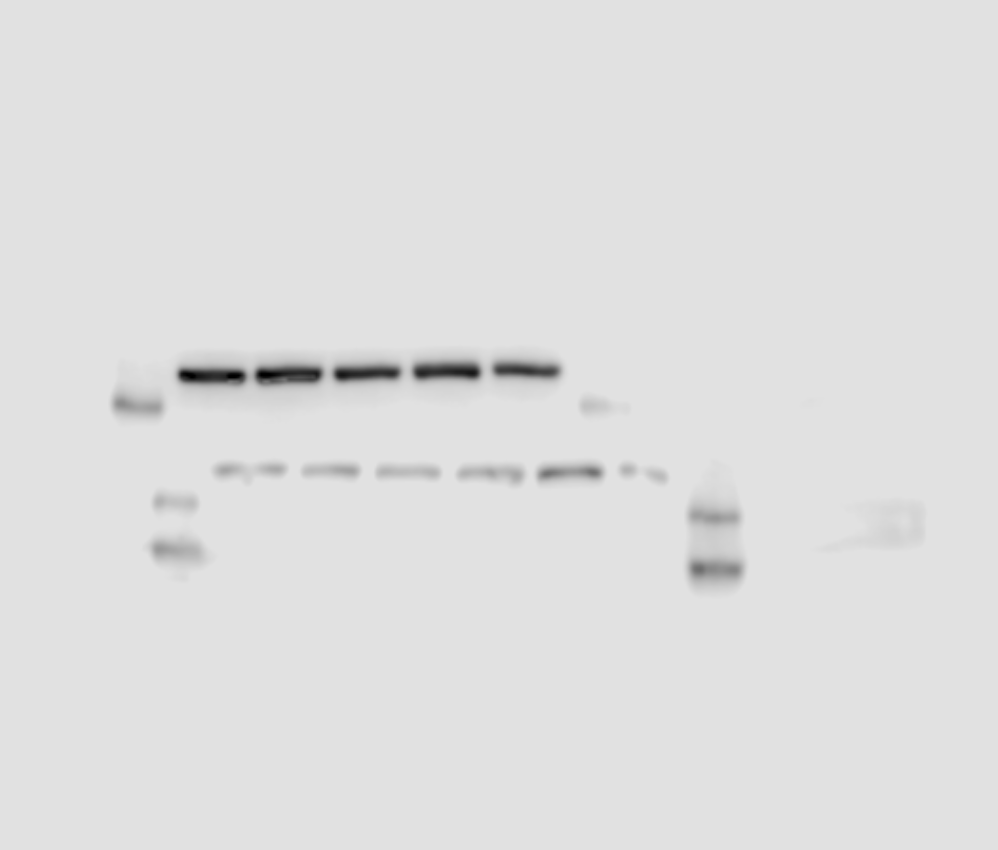

Supplement: Supplementary file 1 [file biomolecules-15-01046-s001.zip › Raw data fig.4 /FIGURE 4D/U-2OS/Actin U-2OS.tif]

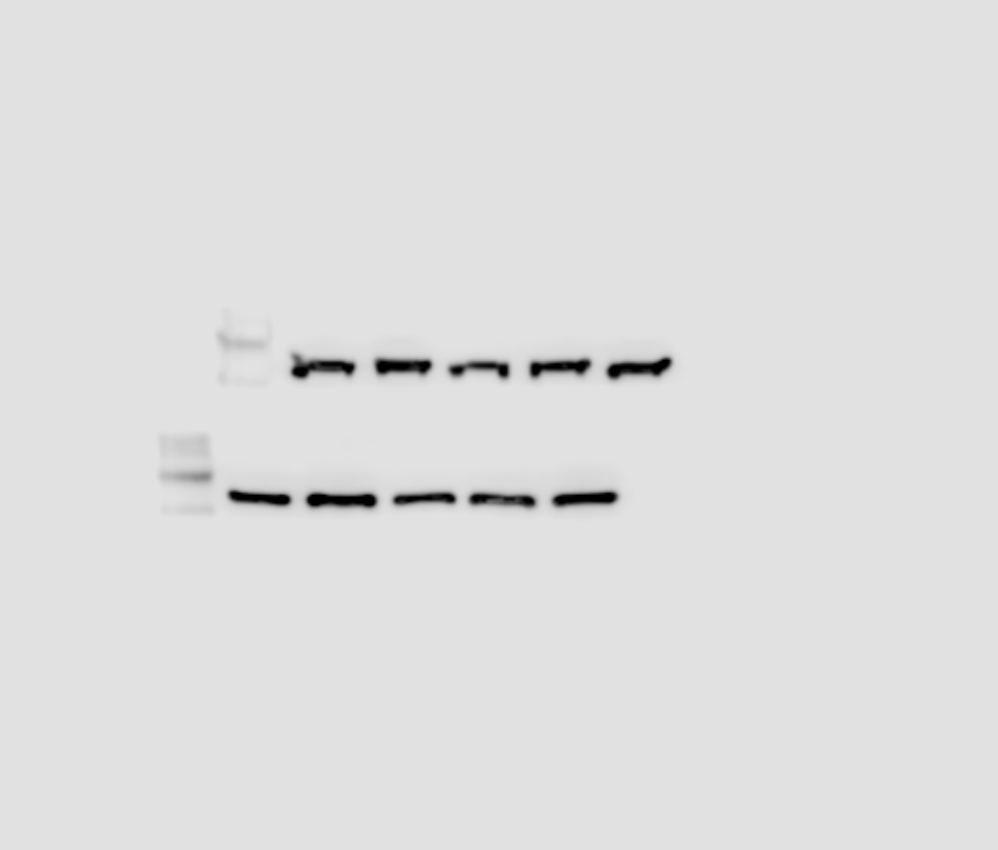

Supplement: Supplementary file 1 [file biomolecules-15-01046-s001.zip › Raw data fig.4 /FIGURE 4B /ACTIN/BUB1 ACTINA2.tif]

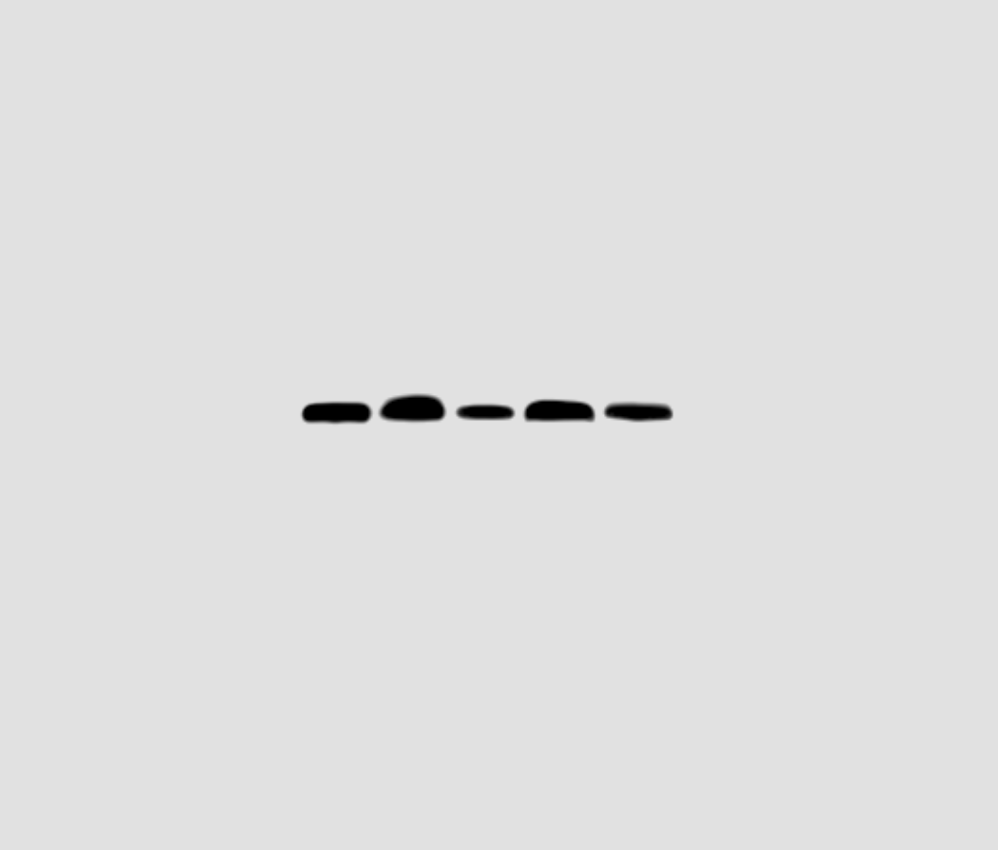

Supplement: Supplementary file 1 [file biomolecules-15-01046-s001.zip › Raw data fig.4 /FIGURE 4B /ACTIN/BUB1 ACTINA3.tif]

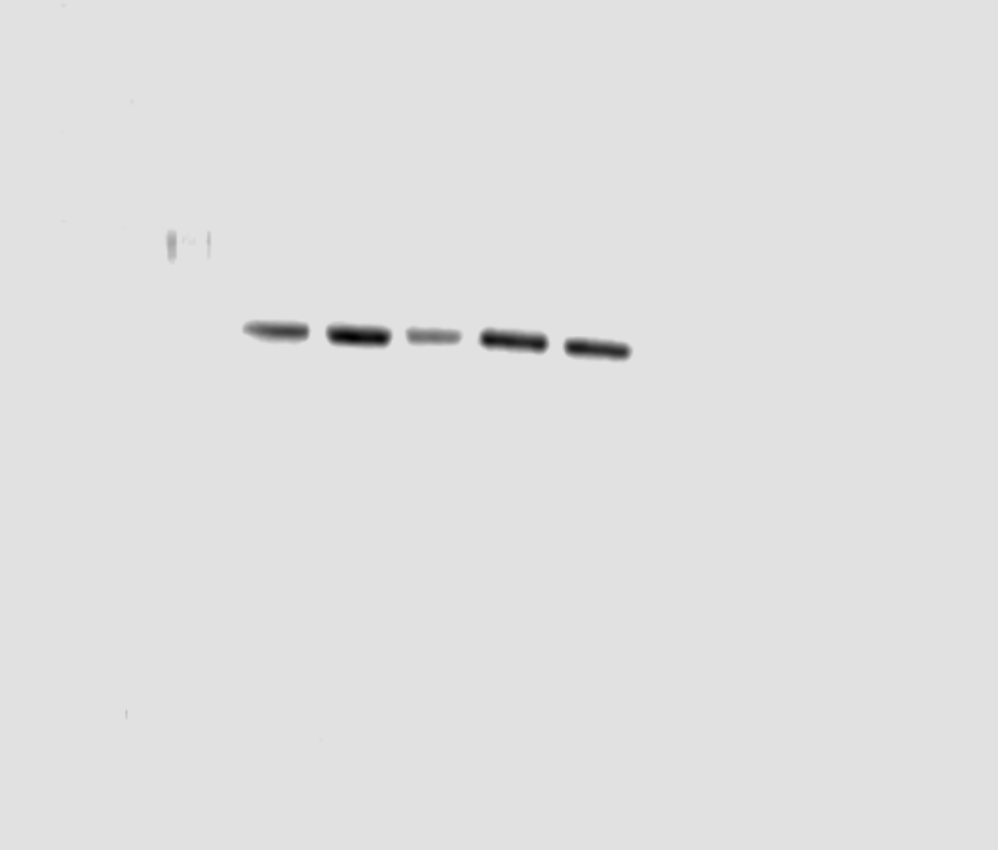

Supplement: Supplementary file 1 [file biomolecules-15-01046-s001.zip › Raw data fig.4 /FIGURE 4B /ACTIN/BUB1 ACTINA1 .tif]

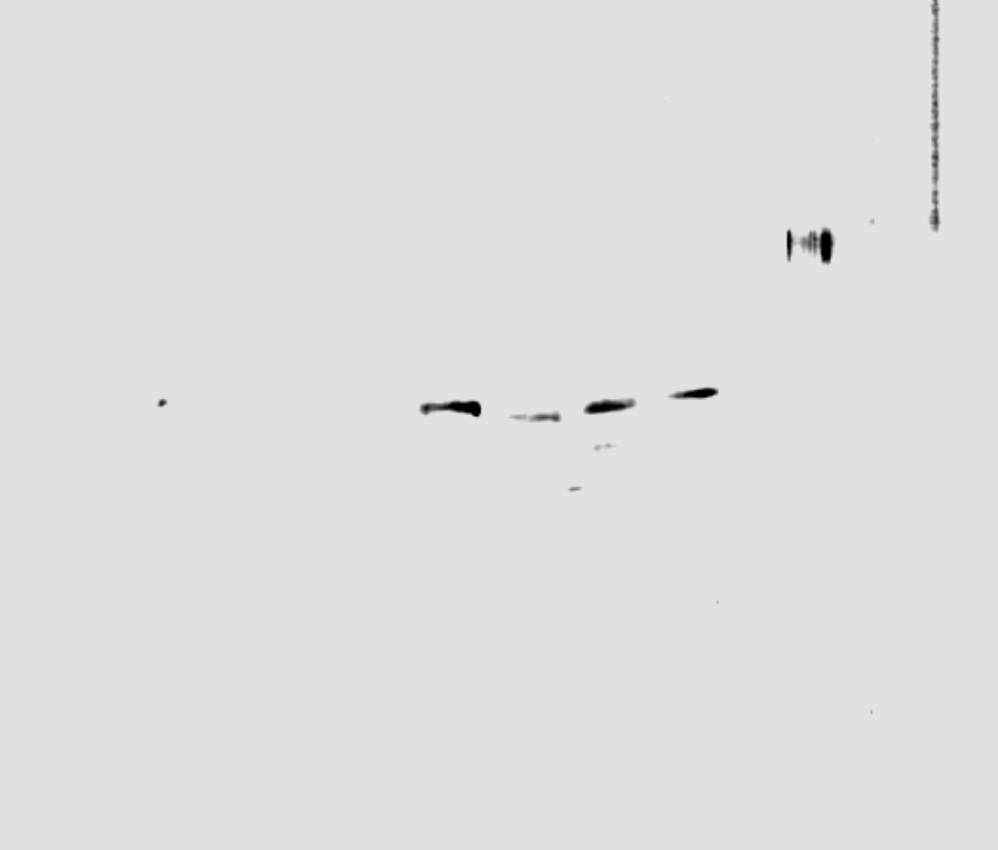

Supplement: Supplementary file 1 [file biomolecules-15-01046-s001.zip › Raw data fig.4 /FIGURE 4B /TOTAL BUB1/TOTAL BUB1 3.tif]

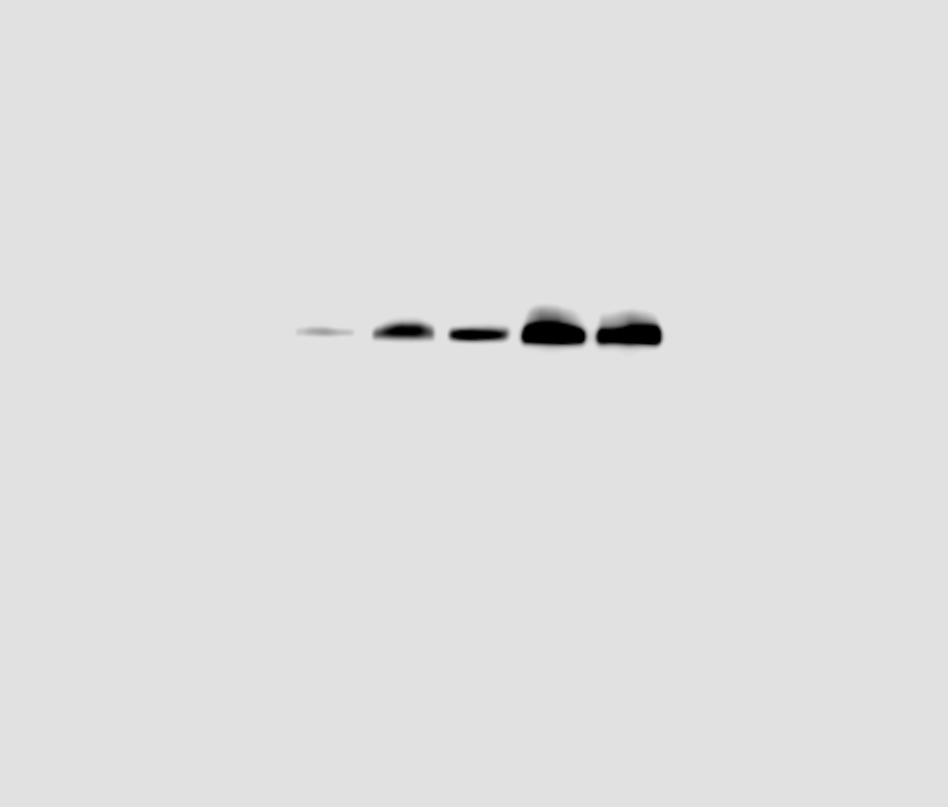

Supplement: Supplementary file 1 [file biomolecules-15-01046-s001.zip › Raw data fig.4 /FIGURE 4B /TOTAL BUB1/TOTAL BUB1 1.tif]

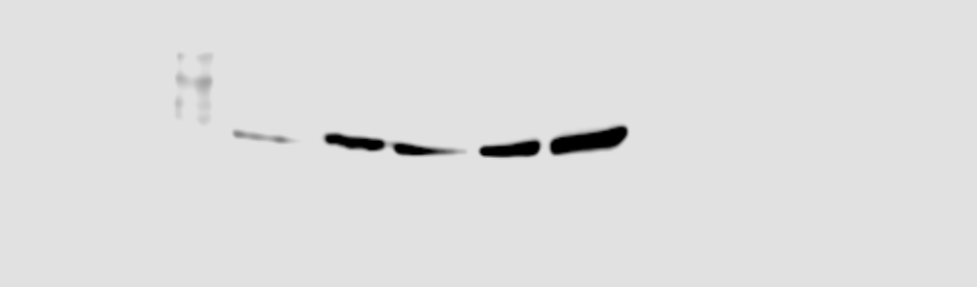

Supplement: Supplementary file 1 [file biomolecules-15-01046-s001.zip › Raw data fig.4 /FIGURE 4B /TOTAL BUB1/TOTAL BUB1 2.png]
